# Robotic‐Assisted Capture‐Systematic Evolution of Ligands by Exponential Enrichment of RNA Aptamers Binding to Small Molecules

**DOI:** 10.1002/cbic.202500264

**Published:** 2025-07-09

**Authors:** Tjasa Legen, Günter Mayer

**Affiliations:** ^1^ Life and Medical Sciences Institute (LIMES) University of Bonn 53121 Germany; ^2^ Center of Aptamer Research and Development (CARD) University of Bonn 53121 Germany

**Keywords:** aptamers, automation, capture‐systematic evolution of ligands by exponential enrichment

## Abstract

Due to their small size, stability, and cost‐effectiveness compared to antibodies, aptamers developed by systematic evolution of ligands by exponential enrichment (SELEX) are promising candidates for the detection of small molecules. In SELEX, a small target molecule is usually covalently immobilized on a surface to separate bound from unbound nucleic acid sequences. However, this immobilization alters the molecule, that is, additional chemical entities are added and the electron distribution is altered, compromising the enrichment properties. To overcome this problem, a capture SELEX method has been successfully developed in which the RNA or DNA libraries are bound to a surface via a complementary oligodeoxynucleotide, and the soluble ligand is used to capture nucleic acids that bind to it from that surface. Herein, the development of an automated version of the capture SELEX method for the identification of RNA aptamers that bind small molecules is described. This method is fully automated and performs up to 12 iterative selection cycles without manual interference in 72 h. The approach is therefore suitable as rapid route to aptamers and enables resource‐efficient test selections to assess “aptamerogenicity” of a target.

## Graphical abstract

This study demonstrates the rapid selection of RNA aptamers that can target various small molecules through a newly established automated capture‐systematic evolution of ligands by exponential enrichment technique. Throughout the study, this method is applied to aptamer selection for several small molecules, resulting in aptamers that bind with high specificity. The affinity of these aptamers is assessed using fluorescence polarization assays and isothermal calorimetry.

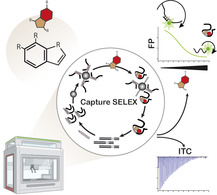

## 1 Introduction

Systematic evolution of ligands by exponential enrichment (SELEX) was introduced as an in vitro technique designed to enrich binding molecules from an oligonucleotide library of ≈10^15^ different sequences. Although the method was introduced as early as 1990,^[^ ^1^, ^2^ ^]^ standardized procedures for selecting aptamers that target a wider range of molecules are lacking. Instead, there are different versions of the procedure, which mainly depend on the used target molecule, the separation methodology, or buffer compositions.^[^ ^3^ ^]^ Besides these in vitro variations, in vivo selection methods were developed that make use of mouse models to target tissue.^[^ ^4^, ^5^ ^]^ In this regard, a plethora of variants of the selection procedure have been established, that are effective in selecting aptamers that bind to different target molecules. However, these methods are hardly comparable in their results, as they were often revised from experiment to experiment and the conditions within a single SELEX experiment were adapted. Therefore, streamlining the SELEX process is an important task that would allow the results to be compared and, if done quickly, the targeting accuracy of a molecule with aptamers to be determined rapidly. To achieve this, we established automated and standardized selection procedures of aptamers binding to proteins.^[^ ^6^, ^7^ ^]^ We focused on targeting small molecules and to avoid problems associated with target immobilization on solid supports, we choose the capture SELEX method.^[^ ^8^ ^]^ The main steps of capture SELEX are immobilization of RNA libraries to magnetic beads using a complementary oligodeoxynucleotide (ODN), the elimination of weakly bound sequences, the recovery of binding sequences by the addition of the small target molecule, followed by the amplification and in vitro transcription of the enriched sequences. For efficient automatization, each step had to be specifically tailored and optimized for a robotic platform. We describe the development of an automated SELEX procedure and its application for the selection of aptamers that target several small molecules. We evaluated different strategies for selecting aptamers using the test target neomycin B and used the most effective strategy to select aptamers for various small molecules. The developed process is fast and enables the high‐throughput identification of RNA aptamers that bind to small molecules.

## 2 Results

### 2.1 Optimization and Adaptation of Capture SELEX for Use on the Robotic Platform

**Figure 1:**
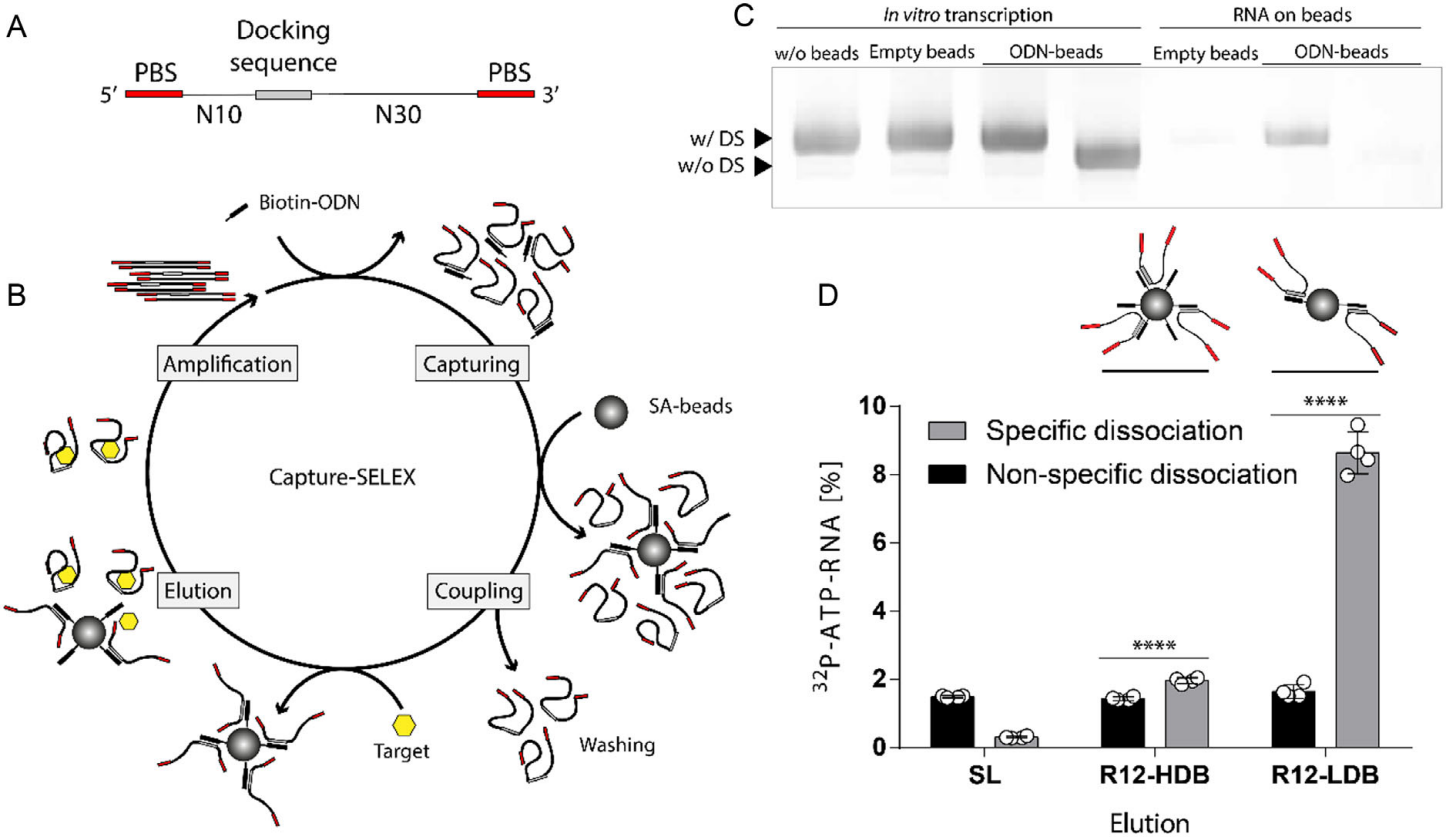
Capture SELEX optimization for automated selection of RNA aptamers targeting small molecules. A) Scheme of RNA library flanked by PBS and a constant 12‐nt DS positioned between N10‐ and N30‐long random region. B) Scheme of RNA capture SELEX. Main steps are 1) capturing of RNA to short oligodeoxynucleotides (ODN) during transcription, 2) coupling of RNA‐ODN duplexes on magnetic beads and removing of weakly bound sequences, 3) elution of binding sequences by addition of a target solution, and 4) reverse transcription and amplification (RT‐PCR) of dissociated sequences. SA: streptavidin. C) RNA library with or without DS was either transcribed in the absence of beads, presence of empty beads, or presence of ODN‐beads complexes complementary to the DS. RNA captured onto the beads was eluted by 8 M Urea and it is depicted with RNA on beads on the gel. RNA can be transcribed and captured to the ODN‐beads, with almost no unspecific interaction. D) Radioactive binding studies with the round 12 library (R12) of neomycin B selection using HDB selection or LDB selection compared to the SL. Captured RNA was either eluted with the selection buffer (nonspecific dissociation) or with 1 mM of neomycin B (specific dissociation). Scheme of the difference of HDB versus LDB is shown above the bars. The selection with LDB shows much greater and specific binding. Error bars show standard deviation (*n* = 2). Unpaired *t*‐test *p* < 0.0001.

In capture SELEX, the RNA library is immobilized on a matrix by hybridization to a complementary oligodeoxynucleotide (capture‐ODN) with a docking sequence embedded in the RNA library (**Figure** **1**A). Sequences that bind to a target molecule and undergo conformational changes can no longer hybridize to the capture‐ODN and are therefore recovered, amplified by reverse transcription (RT)‐polymerase chain reaction (PCR), and used in subsequent selection cycle after transcription into RNA (Figure 1B). We began the implementing capture SELEX in an automated process by using libraries generated from primer binding sites previously used in automation^[^ ^6^, ^9^, ^10^ ^]^ and docking sequences used in capture SELEX studies (Figure 1A).^[^ ^8^ ^]^ In our design, the docking sequence is flanked by a 10 nt upstream and a 30 nt downstream random region (Figure 1A).

We have set up our robotic platform to eliminate the need for manual handling during the enrichment procedure. This eliminates the nucleic acid purification steps that are usually carried out in manual capture SELEX experiments. To implement this concept, we transferred the RNA‐target complexes obtained during the SELEX process directly into the RT‐PCR reaction. After amplification, an aliquot of the unpurified PCR products was used as a template for transcription in vitro.^[^ ^6^ ^]^ To optimize the process, we added magnetic streptavidin beads modified with capture‐ODNs to the transcription mix. As the RNA is transcribed, it binds to the capture‐ODNs on the beads. We analyzed whether the addition of beads without or coupled with capture‐ODNs affected the transcription efficiency, but no noticeable difference in RNA yield was observed in the presence or absence of the beads (Figure 1C). This effect was also independent of the presence of a docking sequence (DS) in the RNA library (Figure 1C). After removal of the reaction solution, we thoroughly washed the beads to eliminate nonspecifically bound RNA. Subsequently, the captured RNA was recovered from the beads with 8 M urea, and the RNA obtained was compared using denaturing polyacrylamide gel electrophoresis (PAGE) (Figure 1C). Our results showed that the RNA binds specifically to the capture‐ODNs on the beads and that only small amounts of RNA bind to streptavidin beads without capture‐ODN as well as only small amounts of RNA without a docking sequence bind to capture‐ODN‐beads (Figure 1C).

### 2.2 Manual Capture SELEX with High‐ and Low‐Density Capture‐ODN Beads

We first tested two different capture SELEX approaches, which differ in the way the RNA library is captured on the beads. In the first approach, the beads were saturated with capture‐ODNs before the RNA library was immobilized.^[^ ^8^ ^]^ In the second approach, the RNA library and capture ODNs were incubated in solution in equal amounts and then immobilized on the beads.^[^ ^11^ ^]^ The main difference between these two methods is that in the first approach, more residual free ODNs remain coupled on the beads because the spherical shape of the beads prevents complete hybridization with RNA. In the second approach, the amount of capture‐ODNs on the beads that are not bound to RNA is low. We named the two methods “high‐density beads (HDB) selection” and “low‐density beads (LDB) selection” and performed twelve selection cycles with neomycin B as target molecule, since it is known to be suitable for capture‐SELEX.^[^ ^12^ ^]^ After 12 selection cycles, we evaluated the binding of the enriched libraries using Cherenkov measurement and ^32^ P‐labeled RNA. The specific recovery of bound sequences by adding neomycin B [1 mM] was observed and compared with the values of the starting library. In addition, we analyzed the nonspecific sequence recovery using a buffer without neomycin B (Figure 1D). Remarkably, the nonspecific sequence dissociation remained consistent across all three libraries (Figure 1D). However, specific recovery increased slightly for the HDB‐enriched and strongly for the LDB‐enriched RNA library. The enriched libraries also showed an increase in capture properties regardless of the method used (Figure S1, Supporting Information).

### 2.3 Robotic Capture‐SELEX

**Figure 2:**
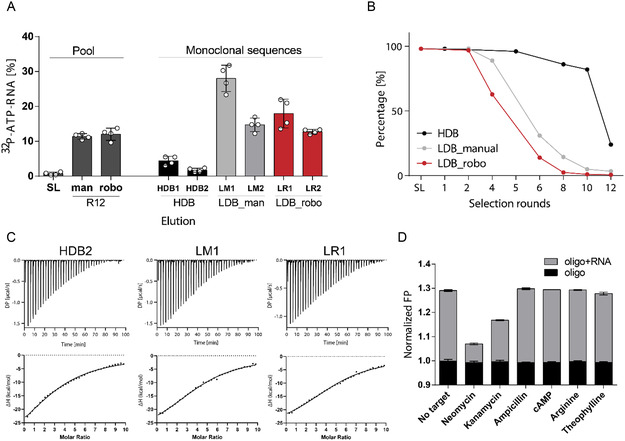
NGS analysis and comparison of different selection strategies for neomycin B. A) Radioactive binding studies of monoclonal sequences enriched through different capture‐selection strategies compared to round 12 of manual and robotic selection. The first two most enriched sequences were chosen in each selection strategy. Applied target concentration was 1 mM. Error bars show standard deviation (n = 2). B) Graph of unique sequences in different selection rounds. The sequences are enriched later in the selection with HDBs as in the selection with LDBs. The enrichment rate of sequences in manual versus robotic (robo) selection is almost equal. C) Affinity of aptamers HDB1, LM1, and LR1 toward neomycin B target measured by ITC. *K*_*d*_ of HDB2, LM1, and LR1 aptamers is 55.9 ± 3.57 μM, 38.0 ± 2.10 μM, and 34.7 ± 1.97 μM, respectively. 1 mM of neomycin B was titrated to 20 μM of the respective aptamer. D) Specificity of the LR1 aptamer measured by FP. The aptamer binds to neomycin B and kanamycin B, but not to other targets: ampicillin, cAMP, theophylline, and L‐arginine. Target concentration is 100 μM. The concentration of capture‐ODN and aptamer LR1 was 100 nM and 500 nM, respectively. Error bars show standard deviation (*n* = 3).

Due to its more pronounced enrichment, we chose the LDB variant for implementation on the robotic platform to select RNA aptamers binding to small molecules. The first automated capture SELEX was performed using neomycin B. Analysis of the PCR products from each selection cycle showed a constant amplification behavior without significant occurrence of by‐products (Figure S2, Supporting Information). All selections followed the same stringent conditions, as described in Table 1, Supporting Information. Interaction analysis of the enriched library shows enhanced binding to neomycin B (**Figure** **2**A). We performed next‐generation sequencing (NGS) of both manual (Figure 1D) and the robotic capture SELEX. The number of unique sequences in the LDB selections showed a similar trend, whether performed manually or automated. The HDB‐variant evolved more slowly and to a lesser extent (Figure 2B). The nucleotide distribution of the RNA from the 12th selection cycles shows the accumulation of different patterns, but is less pronounced in the manual LDB selection (Figure S3, Supporting Information). Among the 20 most abundant sequences in all three selections, only one sequence appeared in all three selections, while two sequences were shared by the LDB variants (Figure S3A, Supporting Information). Motif analysis across all three SELEX experiments revealed two motifs specific to the HDB strategy only and three motifs found common to all strategies, underlining the reproducibility of performance (Figure S4B,C, Supporting Information).^[^ ^13^ ^]^

Next, we evaluated the binding properties of the most enriched sequences from all three SELEX experiments (Figure S4D, Supporting Information). We selected the two most enriched sequences from each selection and, by this test, covered all motifs and sequences found in all three selections or both LDB selections (Figure S4, Supporting Information). Although the two most enriched sequences in HDB selection, HDB1 and HDB2, had a high frequency (10.4% and 10.0%, respectively), they displayed low binding intensities according to Cherenkov measurements (Figure 2A, Figure S4, Supporting Information). In contrast, the most enriched sequences from the manual LDB (LM1 and LM2; 9.8% and 7.9%, respectively) and the robot‐assisted LDB (LR1 and LR2; 33.7% and 26.9%, respectively) showed a stronger binding (Figure 2A, Figure S4, Supporting Information).

The interaction of the sequences with neomycin B and the approximate kinetics of binding were further analyzed by using a fluorescence polarization assay, as previously described in Legen and Mayer.^[^ ^14^ ^]^ The assay utilizes the hybridization of fluorescently labeled short ODNs to the RNA docking sequence, which increases the polarization. Conversely, RNA bound to the ligand when present in solution, results in decreased polarization. After initial assay optimization, the binding of various enriched RNA libraries to neomycin B, that is, starting library (SL), and the enriched libraries from HDB, LDB manual, and LDB robotic selections, and an improved LDB selection later discussed in section. Automated capture selection for different ligands was tested using 100 μM of the neomycin B target in the solution. Figure S5A, Supporting Information showed a decrease in fluorescence polarization for the RNA libraries from the selections performed with LDB as opposed to HDB, consistent with the results from Cherenkov measurements (Figure 1D and Figure 2A). In addition, binding of monoclonal sequences selected for neomycin B was examined. Aptamers LM1, LM2, LR1, and LR2 showed strong binding to the target, while HDB1 and HDB2 showed weak or no binding, respectively (Figure S5B, Supporting Information), consistent with the results from the radioactive binding assay (Figure 2A).

To increase the sensitivity of the assay for low‐affinity binding aptamers and to increase the dynamic range, the stability of the ODN‐RNA duplexes was adjusted. ODNs of different lengths from 8 to 12 nucleotides or an ODN with a point mutation were used. The annealing efficiency of different ODN lengths was observed, with all ODNs annealing efficiently to the LM1 aptamer except the 8‐nt ODN (Figure S5C, Supporting Information). Subsequently, the dynamic range of different ODN lengths that annealed efficiently to the LM1 aptamer was examined, with the 9‐nt ODN showing the highest fluorescence polarization change between free RNA and RNA with the target (Figure S5D, Supporting Information).

Finally, we used shortened, fluorescently labeled ODNs to titrate different concentrations of neomycin B to aptamers HDB1, LM1, and LR1 (Figure S6A, Supporting Information), and determined the approximate affinity to be 10 μM, 4 μM and 3 μM, respectively. Furthermore, we designed a scrambled sequence of LM1 aptamer (ScrLM1) to show that the aptamers’ binding is sequence‐specific. In fact, as shown in Figure S6B, Supporting Information, we observed no detectable decrease in fluorescence polarization when neomycin B was added to the capture ODN and ScrLM1 hybrid. Next, we utilized isothermal titration calorimetry (ITC) to confirm and more accurately determine the dissociation constants (*K*_*d*_). Due to the elevated differential power (DP) values observed during the titration of neomycin B in buffer (Figure S6C, Supporting Information), we subtracted the heat generated by ligand titration in buffer from the heat generated by the titration of the ligand to the respective aptamers (Figure 2C). This time, we examined the affinity of the HDB2 aptamer instead of HDB1, since we wanted to determine the detection limit for affinity that could not be identified using radioactive or fluorescence polarization assays (Figure 2A, Figure S5B, Supporting Information). The *K*_*d*_‐value for all three aptamers was determined in the micromolar range (HDB2: 55.9 ± 3.57 μM, LM1: 38.0 ± 2.10 μM, and LR1: 34.7 ± 1.97 μM, Figure 2C).

In addition, the LR1 aptamer was found to bind to neomycin B and kanamycin B when used in a fluorescence polarization assay, but did not interact with ampicillin, cAMP, arginine, or theophylline (Figure 2D). Therefore, we examined the binding affinity of the LR1 aptamer to kanamycin B. The heat generated by kanamycin B titration in the buffer was again subtracted from kanamycin B titration in the LR1 aptamer solution (Figure S6D, Supporting Information). ITC measurements revealed that the LR1 aptamer had an affinity for kanamycin B of 130.0 ± 30.9 μM, which is almost four times lower than the affinity of LR1 for neomycin B (Figure S6E, Supporting Information).

### 2.4 Automated Capture Selection for Different Ligands

After having established an automated capture SELEX process, we extended the approach to other small molecule targets. In this context, we used L‐arginine and theophylline, which had previously been used in the selection of RNA aptamers.^[^ ^15^, ^16^ ^]^ Unfortunately, after the fourth round of automated SELEX, the PCR profiles showed a shortening of the DNA, indicating an unsuccessful enrichment process and the occurrence of so‐called molecular parasites (Figure S7, Supporting Information). Our hypothesis for the shortening of the DNA was that the initially high target concentration in the transcription mix (90 μM) prevented the transcribed RNA molecules from binding to the capture ODN and instead favoring their binding to the target. As a result, only sequences that dissociated nonspecifically were advanced to the next selection step. These sequences, which did not need the random region for their evolution, could have lost it to amplify more efficiently. This phenomenon did not occur with neomycin B, probably because its binding ability was weakened by the repeated heating and cooling cycles during PCR and prolonged incubation at 37 °C during in vitro transcription reaction.^[^ ^17^ ^]^ Although all three molecules are relatively stable at 95 °C, repeated heating and cooling can lead to degradation over time. Of the three, theophylline is the most stable up to about 270 °C, and retains much of its structure with moderate temperature variations.^[^ ^18^ ^]^ L‐arginine starts to degrade at about 180 °C,^[^ ^19^ ^]^ while neomycin B can degrade at slightly lower temperatures with repeated heat exposure,^[^ ^20^ ^]^ making neomycin B less thermally stable than theophylline and arginine, which could be the cause for observed selection failure. Of note, lowering the initial concentration of neomycin B to 50 μM and 5 μM or performing 12 selection cycles without the target resulted in a degree of DNA truncation similar to that seen in other unsuccessful selections (Figure S8, Supporting Information).

**Figure 3:**
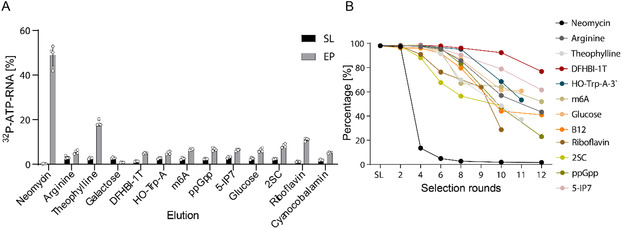
RNA capture SELEX with different small molecule targets. A) Binding comparison of enriched pool (EP) of different small molecule targets. Applied target concentration was 0.1‐1 mM. Error bars show standard deviation (*n* = 2). B) Graph of unique sequences in different selection rounds by the selections with different small molecule targets measured by NGS. DFHBI‐1 T: 3,5‐Difluoro‐4‐hydroxybenzylidene imidazolinone‐1 T,^[^ ^39^ ^]^ HO‐Trp‐A: D‐Tryptophanyl Adenosine 5′‐Monophosphate Phosphoramidate,^[^ ^37^ ^]^ m6A: N6‐Methyladenosine, ppGpp: Guanosine pentaphosphate, 5‐IP7: 5‐diphospho‐myo‐inositol pentakisphosphate,^[^ ^21^ ^]^ 2SC: S‐(2‐succinyl) cysteine.

Based on these observations, we modified the automated capture‐SELEX procedure and purified the PCR product from the target before proceeding to the transcription step. To do this, we separated the RT from the amplification step. In the optimized version, RT occurred in the presence of a biotinylated reverse primer and immobilization of the thus biotinylated cDNA to streptavidin‐coated magnetic beads (Figure S9, Supporting Information). The supernatant containing the target molecule was then removed and the PCR mix was added to amplify the immobilized cDNA. With the optimized method, the truncation of DNA during SELEX was reduced, although shorter bands occasionally appeared, but they never dominated the library. Remarkably, these by‐products did not reappear after reamplification or visualization by denaturing gel electrophoresis, suggesting they could be secondary structures or single‐stranded DNA (Figure S10, Supporting Information). We applied the optimized capture‐SELEX scheme to 13 target molecules that varied in chemical structure and functionality (**Figure** **3**A, Figure S11, Supporting Information). Cherenkov measurements of the enriched libraries showed binding to neomycin B, theophylline, and riboflavin, and to a lesser extent to all other targets except galactose, where the amplified band weakened after round six (Figure S12C, Supporting Information).

NGS analysis of the enriched libraries showed a strong decline of unique sequences for the selection using neomycin B after the fourth selection cycle, whereas for the other targets, a decline in unique sequences only began after the sixth round, and to a much lesser extent (Figure 3B).

### 2.5 NGS Analysis and Interaction Properties of Monoclonal Sequences

After NGS analysis, we took a deeper look at the enrichment profiles of each selection and its most enriched sequences. We found that several identical sequences were enriched during the course of selections, which target different small molecules (Figure S13, Supporting Information). However, the frequency of these sequences in the enriched libraries was close to zero until the fourth cycle of selection, and most sequences only began to accumulate after the sixth cycle. Some of the sequences (i.e. 1, 3, 4, 6, and 7) were tested for binding, but none of them showed binding to the tested targets (data not shown). We hypothesized that for the selections with little enrichment success, well‐amplifying sequences were preferentially enriched, independently of the target used, so that the enriched sequences were identical for selections targeting different ligands.

Next, we searched for the most promising sequences in all selections and examined their binding. As shown in Figure S14, Supporting Information, the 100 most enriched sequences were sorted into different families with no more than 5 point mutations (PM) (only up to six families or 18 families were presented in the case of ppGpp). In addition, common motifs among the 100, the most enriched sequences were identified using the MEME suite tool for motif discovery.^[^ ^13^ ^]^ In some selections, specific motifs were found and at least one representative sequence of each motif was tested for the binding. In other cases, two to three of the most enriched sequences were tested, trying to avoid sequences that were frequently enriched in several different selections (Figure S13, Supporting Information). Remarkably, the most enriched sequence in the optimized selection approach targeting neomycin B was identical to the HDB12, LM1, and LR5 sequence, which was frequently enriched in all neomycin B selections (Figure S4,S14, Supporting Information).

**Figure 4:**
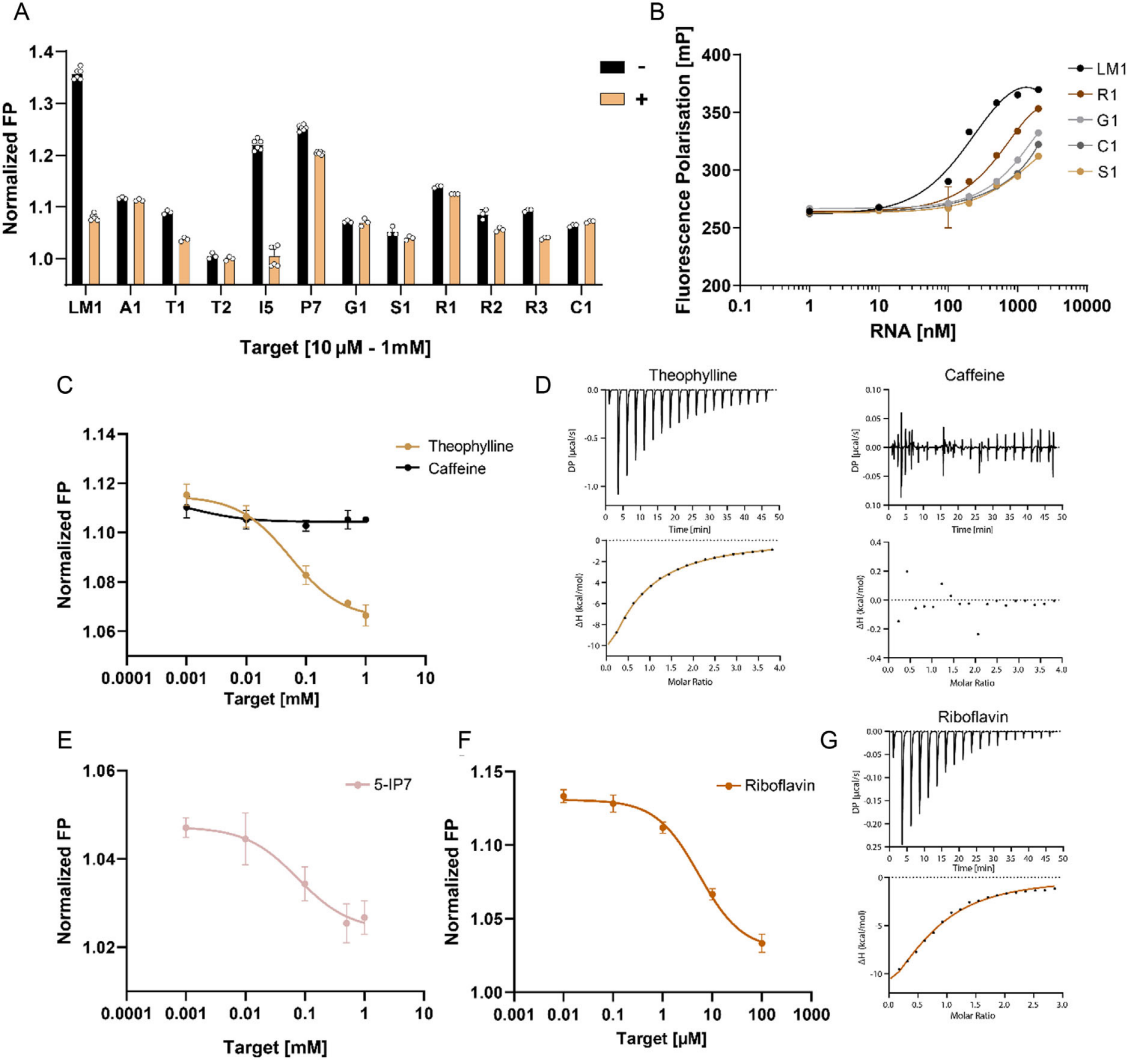
Characterization of monoclonal sequences targeting various small molecule ligands. A) Binding test of various monoclonal sequences targeting ligands such as neomycin B (LM1), arginine (A1), theophylline (T1, T2), 5‐IP7 (I5), ppGpp (P7), glucose (G1), S‐(2‐succinyl) cysteine (S1), riboflavin (R1, R2, R3), and cyanocobalamin (C1). Interaction was measured by FP assay utilizing Cy3‐labeled 9‐nt complementary oligodeoxynucleotide (ODN). In the assay, 100 nM of Cy3‐labeled capture‐ODN and 500 nM RNA were used in the absence or presence of target. All values were normalized according to the FP value of Cy3‐labeled capture‐ODN. Error bars show standard deviation (*n* = 3). B) Annealing efficiency of different monoclonal sequences to the corresponding 9‐nt complementary ODN. Efficiency was measured by FP assay. In the assay, 100 nM of Cy3‐labeled capture‐ODN and 500 nM RNA were used. Error bars show standard deviation (*n* = 3). C) Concentration‐dependent binding of theophylline or caffeine to T1 aptamer measured by FP. The estimated *K*_*d*_ of T1 aptamer binding to theophylline determined by the assay was ≈54 μM. The fluorescence change was fitted to inhibitor vs. response model. Error bars show standard deviation (*n* = 3). D) Thermodynamic properties of the interaction of theophylline or caffeine with the T1 aptamer measured by ITC. The determined *K*_*d*_ of T1 aptamer binding to theophylline was 70.3 ± 6.33 μM. 1 mM of theophylline or caffeine was titrated to 50 μM of T1 aptamer. E) Concentration‐dependent binding of 5‐IP7 to I5 aptamer measured by FP. The estimated *K*_*d*_ determined by the assay was ≈76 μM. The fluorescence change was fitted to inhibitor versus response model. Error bars show standard deviation (*n* = 3). F) Concentration‐dependent binding of riboflavin to R3 aptamer measured by FP. The estimated *K*_*d*_ determined by the assay was ≈5 μM. The fluorescence change was fitted to inhibitor versus response model. Error bars show standard deviation (*n* = 3). G) Thermodynamic properties of the interaction of riboflavin and R3 aptamer measured by ITC. The determined *K*_*d*_ was 9.88 ± 1.09 μM. 300 μM of riboflavin was titrated to 20 μM of R3 aptamer.

The interaction of representative sequences from the selections made (marked in Figure S14, Supporting Information with bold) was tested using the fluorescence polarization assay. Among these sequences, LM1 and I5 from the neomycin B and 5‐IP7^[^ ^21^ ^]^ selections, respectively, showed the strongest binding, while T1, P7, R2, and R3 showed weaker binding to theophylline, ppGpp, and riboflavin, respectively (**Figure** **4**A). The remaining tested sequences that did not bind are not shown in the graph. As seen in Figure S15, Supporting Information, different RNA libraries exhibited varying annealing efficiencies toward the capture‐ODN. Furthermore, in Figure 4B, monoclonal sequences with identical docking sequences were found to bind to capture‐ODNs with different affinities. We then attempted to determine the dissociation constant (*K*_*d*_) of two binding sequences by the fluorescence polarization method and compared these values with those obtained by ITC measurement. Figure 4C shows that aptamer T1 binds to theophylline with a measured *K*_*d*_ of 54 μM, with no binding observed to the structural analog caffeine. Likewise, ITC‐measured interaction of T1 and theophylline yielded a *K*_*d*_‐value of 70.3 ± 6.33 μM, with no interaction observed for caffeine (Figure 4D). Similarly, Figure 4E shows concentration‐dependent binding of 5‐IP7 to aptamer I5, resulting in a calculated *K*_*d*_ *‐*value of 76 μM. We attempted to determine the binding affinity of the I5 aptamer using ITC, but the experiment was unsuccessful due to the precipitation of the target at high concentration. Finally, we tested the interaction of riboflavin and aptamer R3 by fluorescence polarization, which resulted in a *K*_*d*_ *‐*value of 5 μM, and the interaction measured by ITC yielded a value of 9.88 ± 1.09 μM (Figure 4 G).

## 3 Discussion

In our study, we sought to automate the selection of RNA aptamers that bind small molecules by applying capture SELEX. Our initial focus was on gaining a thorough understanding of the various capture SELEX methods described in previous work and using this information to adapt the process to an automated format.^[^ ^8^, ^11^ ^]^ The results of our experiments indicated that LDB selection resulted in sequence enrichment with stronger recovery of RNA in the presence of neomycin B compared to HDB selection (Figure 1D). This difference in specific recovery of RNA species clearly demonstrates that LDB selection outperforms HDB selection and that the density of capture‐ODNs on the beads plays an important role in the enrichment of RNA libraries during the capture SELEX procedure. We hypothesize that the reason for the lower efficiency of HDB selection is that numerous free ODNs remain on the beads, causing the captured RNA to bind to the next available ODN instead of dissociating and binding to the target. This assumption is consistent with the fact that target molecules prevent the RNA from rebinding to the capture‐ODNs on the beads after dissociation, rather than playing a more active role in destabilizing the duplex structure between the capture‐ODN and the docking sequence in the RNA library. Furthermore, we have successfully applied this methodology to the robotic platform and performed it using neomycin B as a model target.

To characterize the enriched sequences of all three different selections (manual and automated) with neomycin B in more detail, we performed NGS analysis and compared the most enriched sequences. Although we used the same library and the same target, we enriched different sequences in all three selections. Nevertheless, some motifs and one sequence were retained in all three selections (Figure S4, Supporting Information). Since we did not find many dominating motifs in the enriched libraries (Figure S4B, Supporting Information), we decided to test the binding of the first two most enriched sequences in each of the selections, where at least one sequence contained the motifs one to four (Figure S4, Supporting Information) and both sequences were present in either all three selections or both LDB selections. All six sequences showed binding to neomycin B, but the HDB1 and HDB2 sequences, both of which were enriched in HDB selection, showed very weak binding. To confirm these results and determine the *K*_*d*_ for subsequent ITC measurement, we performed target‐dependent displacement of a representative aptamer (HDB1, LM1, and LR1) from fluorescently labeled capture‐ODNs using different concentrations of neomycin B (Figure 2, Figure S6A, Supporting Information). The affinities determined were 4 μM and 3 μM for aptamers LM1 and LR1, respectively, which were about ten times lower as the results obtained by ITC measurement (38.0 ± 2.10 μM and 34.7 ± 1.97 μM for LM1 and LR1 aptamer, respectively).

However, the affinities obtained were lower than those previously reported for neomycin B‐binding RNA aptamers (*K*_*d*_ ≈ 100 nM),^[^ ^12^ ^]^ which were selected using an immobilized target. This condition could allow for more stringent separation of binding and nonbinding sequences. In accordance with this notion are results of selections with the capture SELEX method, which targets structurally similar aminoglycoside antibiotics and resulted in micromolar affinity DNA aptamers.,^[^ ^8^ ^]^ but low micromolar affinity has also been reported in other capture SELEX aptamer publications.^[^ ^22^, ^23^, ^24^, ^25^, ^26^, ^27^ ^]^ This could be due to the nature of the small molecule targets, which have fewer functional groups with which to interact. However, aptamers with much stronger affinities have been reported in studies using immobilized small molecule targets.^[^ ^28^, ^29^, ^30^ ^]^ Nevertheless, in some published studies using the capture SELEX method aptamers have been obtained that bound to their ligands in the nanomolar range.^[^ ^31^, ^32^, ^33^ ^]^ Most of these studies describe well‐designed and target‐specific selection procedures that take into account the initial target concentration and usage of several structurally similar analogs for counter‐selection in their selection approach.^[^ ^32^, ^33^ ^]^ Alkhamis and Xiao^[^ ^33^ ^]^ performed several selection experiments with flunixin. Their results suggest that using lower initial target concentrations and reducing its concentration very sharply during the selection could lead to an enrichment of aptamers with stronger affinity. They also employed a well‐thought‐out counter‐selection strategy, demonstrating its importance.

We also applied the automated selection strategy to select aptamers that target other ligands (Figure 3). Although we were able to enrich aptamers for some ligands, all tested aptamers still bind in the micromolar range (Figure 4). Therefore, we believe that adjusting the ligand concentration specifically for each target could improve the affinity of selected candidates and increase the overall success rate. The high‐throughput platform would also allow several selection experiments to be carried out simultaneously for each target, since, as we have also shown in Figure S8, Supporting Information, if the starting target concentration is too low, this could lead to the loss of binding sequences and to the appearance of selection artifacts, for example, truncation of the library in the early selection cycles. To improve the specificity of selected aptamers, one could introduce additional elution with counter targets or add the counter targets to the washing buffer solution, although neomycin B and theophylline binding aptamers selected with the current method show fairly specific interactions with respective targets (Figure 2A and Figure 4C,D). Another way to improve the affinity of enriched aptamers would be to gradually extend the capture‐ODN:RNA complex by using longer capture‐ODNs in the selection procedure. This way, ligand interaction would have to prevent rebinding of RNAs mediated by longer base‐pairing sites, thus directing selection toward more tightly binding aptamers. The studies mentioned above, which yielded aptamers in the nanomolar range, also used the position of the docking sequence at the 5′ end of the library.^[^ ^32^, ^33^ ^]^ This could also contribute to the enrichment of aptamers with higher affinity. However, no studies have yet been carried out in which the length and position of the docking sequence and its effects on the enrichment of high‐affinity binding aptamers are systematically investigated. We therefore believe that our platform, which allows multiple selection processes and is operator‐independent, enables deeper exploration and a better understanding of the SELEX process.

We also presented a quick and easy fluorescence polarization assay that serves as a cost‐effective alternative to time‐consuming radioactive binding assays because it does not require RNA labeling and expensive magnetic beads. A further advantage was the possibility of automation with spectrophotometric plate readers attached to the pipetting station. Using enriched libraries and monoclonal sequences for neomycin B, we optimized the length of the capture‐ODN that produced the greatest change in fluorescence between the absence and presence of the ligands (Figure S5, Supporting Information). Shortening of the capture‐ODN to 9‐nt still ensured that the RNA bound well to the ODN, but at the same time also prevented the rebinding of the RNA to the capture‐ODN in the presence of the ligand. We used the same length of the capture‐ODN to test the binding of other monoclonal sequences targeting different ligands (Figure 4A), but for some sequences we observed very weak binding to the capture‐ODN. We tested the concentration‐dependent annealing of several monoclonal sequences, and although all sequences had the same complementary docking sequence to the capture‐ODN, the annealing kinetics was quite different than for the LM1 aptamer (Figure 4B). Therefore, we believe that a specific adjustment of the aptamer concentration could help to verify binding when a shorter capture‐ODN length is used.

In summary, we have demonstrated a fast and fully automated process for selecting RNA aptamers for small molecules without the need to immobilize the target. Furthermore, we see potential for adapting this procedure to the selection of DNA aptamers or even chemically modified DNA aptamers, for example, clickmers.^[^ ^34^, ^35^ ^]^ In a very recent publication, an automated procedure to gain DNA aptamers binding to a glycoprotein was established.^[^ ^36^ ^]^ In a similar manner, our platform could also be extended to generate peptide‐ or protein‐binding RNA aptamers using the automated capture‐SELEX approach described here. Although our method has proven effective for most targets tested, we anticipate that by further optimization of conditions, such as target concentration and washing procedures, we will increase the success rate of this selection method and enrich high‐affinity aptamers. The automated process allows for rapid screening of aptamer targetability of specific targets, and thus provides a unique approach to stratify targets for further development and selection procedures.

## 4 Experimental Section

4.1

4.1.1

##### Materials

Antibiotics Neomycin B, ampicillin, and kanamycin B were purchased from Roth, Germany. Theophylline, L‐arginine, galactose, S‐(2‐Succinyl)‐L‐cystein (2‐SC), cyanocobalamin, and cAMP were purchased from Sigma‐Aldrich, Germany. D‐Tryptophanyl Adenosine 5′‐Monophosphate Phosphoramidate (HO‐Trp‐A‐3′)^[^ ^37^ ^]^ was provided by Clemens Richerts (University of Stuttgart) and N6‐Methyladenosine (m6A) by Mark Helm (University of Mainz). Guanosine pentaphosphate (ppGpp) and 5‐diphospho‐*myo*‐inositol pentakisphosphate (5‐IP7)^[^ ^21^ ^]^ were provided by Henning Jessen and Andreas Meyer (University of Freiburg and University of Lausanne). N6‐Methyladenine was purchased from Cayman Chemical, USA and DFHBI‐1 T from Bio‐Techne, Germany. Other chemicals were purchased from Roth, Germany.

##### Selection Using HDBs Approach

Oligodeoxynucleotide (ODN) beads were prepared by first washing 10 mg of Dynabeads *M*‐280 Streptavidin (Thermo Fisher Scientific) 3‐times with Binding and Washing buffer (5 mM Tris, 0.5 mM EDTA, 1 M NaCl) and resuspending them in 1 mL of 5 μM ODN solution in BWB buffer (for sequence information see *Manual preparation of RNA‐oligo‐beads complexes for the initial round*). The mixture was incubated on a rotator for 1 h at room temperature. Afterward, ODN‐beads were washed 3‐times with 1 mL of 40 mM Tris buffer pH 7.9 and resuspended in 150 μl. 22.5 μl of prepared ODN‐beads (1.5 mg beads, 600 pmol ODN) were mixed with 750 pmol of RNA library in the final concentration of 40 mM Tris pH 7.9 with 35 mM Mg^2+^ and incubated overnight at 37 °C and 900 rpm (for preparation of RNA library see *Manual preparation of RNA‐oligo‐beads complexes for the initial round*). The rest of the ODN‐beads solution was stored at 4 °C for following rounds. The next day, supernatant was discarded and the beads were washed 6‐times with 200 μl and 3‐times for 5 min with 100 μl of the selection buffer (primer binding sites (PBS), 3 mM Mg^2+^ pH 7.4). Next, 50 μl of 1 mM neomycin B solution in the selection buffer was added to the beads and incubated for 15 min at 21 °C and 900 rpm. After incubation, 45 μl of the eluate was removed from the beads and mixed with RT‐PCR solution (1x GoTaq buffer, 1.35 mM Mg^2+^, 2 mM DTT, 1 μM forward primer, 1 μM reverse primer, 0.3 mM dNTPs, GoTaq Polymerase (Promega)). The program for RT‐PCR is described in the *Automated selection*. After amplification, 10 μl of the PCR product was transferred to the transcription mix (40 mM Tris pH 7.9, 5 mM DTT, 35 mM Mg^2+^, 2.5 mM NTPs, 0.5 mg oligo‐beads, T7‐RNA polymerase (homemade)). The in vitro transcription ran for 1.5 h at 37 °C and 900 rpm. Next, the supernatant was removed and the beads were washed 3‐times for 5 min with 100 μl for of the selection buffer as done in the beginning of the round. Repeated rounds were done in the same way with increasing stringency by decreasing target concentration (1–0.5 mM), decreasing time of incubation of beads with the target solution (15–5 min), and decreasing the transcription time (1.5–0.5 h). The selection was done for 12 rounds.

##### Manual Preparation of RNA‐Oligo‐Beads Complexes for the Initial Round

The design of primer‐binding sites was taken from Breuers et al.^[^ ^6^ ^]^ and 12‐nt DS design from Stoltenburg et al.^[^ ^8^ ^]^ The DS was placed between 10‐ and 30‐nt random region. Library was purchased as single‐stranded DNA from Ella biotech, Germany (5′‐GGGAGAGGAGGGAGATAGATATCAA‐N10‐TGAGGCTCGATC‐N30‐TTTCGTGGATGCCACAGGAC‐3′). Forward primer with T7 promotor (5′‐AATTCTAATACGACTCACTATAGGGAGAGGAGGGAGATAGATATCAA‐3′), reverse primer (5′‐GTCCTGTGGCATCCACGAAA‐3′), and biotinylated 12‐nt capture oligodeoxynucleotide (ODN) (5′‐biotin‐18spacer–GATCGAGCCTCA‐3′) were provided by Ella Biotech, Germany. First, RNA library was prepared by a large‐scale PCR of 10 mL by using 1 nmol of synthetized ssDNA pool. PCR was performed with the program: (1) 95 °C 2 min, (2) 95 °C 1 min, (3) 56 °C 1 min, (4) 72 °C 1 min, and (5) 72 °C 3 min, with repeated steps 2−4 for 5 cycles (1x GoTaq buffer, 1.5 mM Mg^2+^, 1 μM reverse primer, 1 μM forward primer, 0.3 mM dNTPs, 5 U GoTaq polymerase (Promega)). Produced DNA was purified by phenol/chloroform extraction followed by ethanol precipitation. One‐quarter of the DNA was used for 1 mL of in vitro transcription (40 mM Tris pH 7.9, 5 mM DTT, 35 mM Mg^2+^, 2.5 mM NTPs, T7 polymerase (homemade)). The solution was incubated at 37 °C overnight. The next day, the RNA was precipitated by ethanol precipitation and the pellet was resuspended in 4x PAA buffer (5.8 M Urea, 0.1 M EDTA, 10 % glycerol). RNA was separated from other nucleic acids on 10 %urea PAGE electrophoresis and the isolated band was crushed and shaken in 0.3 M NaOAc pH 5.9 for 2 h at 65 °C. Afterward, the RNA solution was sifted through the syringe with glass wool to remove gel particles. Finally, the RNA solution was precipitated by Ethanol precipitation and the RNA was resuspended in ddH_2_O and stored at −20 °C. For the initial round of selection, the RNA was manually captured to the beads. 1.5 nmol of RNA and 1.5 nmol of 12‐nt capture ODN was mixed in 100 μl selection buffer (PBS, 3 mM Mg^2+^ pH 7.4) and left to anneal by applying 50 °C for 15 min and slowly cooling down to 30 °C for 45 min with gentle shaking. In the meantime, 2.5 mg of Dynabeads *M*‐280 Streptavidin (ThermoFisher Scientific, USA) was washed 3‐times with 500 μl selection buffer by DynaMag‐2 Magnet (ThermoFisher Scientific, USA). Finally, the beads were resuspended in 50 μl selection buffer and mixed with RNA‐ODN solution. The mixture was incubated for 30 min at 21 °C with gentle shaking to allow RNA‐ODN complexes to be coupled to the beads. Afterward, the supernatant was discarded and 100 μl of fresh selection buffer was added to the beads.

##### Selection Using LDBs Approach

The selection was done manually in the same way as described in *Manual preparation of RNA‐oligo‐beads complexes for the initial round* with following selection described in *Automated selection*. Neomycin B concentration during the selection was 1−0.05 mM. The amount of Dynabeads *M*‐280 Streptavidin used in selection round 2−12 was 1 mg.

##### Robot Preparation

The selection process was done on Biomek NXp station (Beckman Coulter, USA) including different ALPs positions (heating/cooling devices, freezing device, magnet, and reservoir holder), a gripper, and a spin‐8 pipette. A PCR cycler (Biometra, Germany) and a hotel, where filter tips (Beckman Coulter, USA) were stored, were connected to the Biomek NXp station with a gripper hand and a moving lane. The software, which was used for the programming of the selection process, was Biomek Software 4.1 and SAMI EX 4.1. All reagents were stored at 4 °C and enzymes at −25 °C. Reagents were stored in 96‐well PCR plates (Starlab, Germany) and were sealed with X‐Pierce film (Sigma‐Aldrich, Germany). All selection steps were performed in 96‐well PCR plates (Eppendorf, Germany). The magnetic station included magnetic rings suited for 96‐well plate. To prevent evaporation of the liquid in the PCR cycler, a removable auto‐sealing plate lid was utilized (Biorad, USA). The selection buffer was stored in a reservoir (Beckman Coulter, Germany). Pipettes calibration was done before initiation and after each selection round. The room was climatized to 19 °C to decrease evaporation of the liquid.

##### Automated Selection

Before the start of the selection, all required reagents as target solution, RT‐PCR mix, transcription mix, beads solution, selection buffer (PBS, 3 mM Mg^2+^ pH 7.4, in the case of 5‐IP7 and ppGpp selection, ICB buffer [12 mM HEPES, pH 7.7, 135 KCl, 10 mM NaCl, 3 mM Mg(OAc)_2_] was used), and enzymes were prepared, aliquoted in plates and stored at either 4 °C (reagents), −25 °C (enzymes), or at room temperature (buffer). The whole volume of RNA‐oligo‐beads solution (described in *Manual preparation of RNA‐oligo‐beads complexes for the initial round*) was transferred to the incubation plate and placed to the Biomek NXp pipetting station. The solution was first incubated for 5 min at 21 °C with gentle shaking. Then, the plate was moved to the magnet position and allowed beads for 2 min to be moved to the magnet. The solution was discarded and 100 μl of fresh selection buffer was added to the beads. Plate was moved to the standard position and the beads were pipetted several times up and down. The plate was again moved to the magnet position and the same procedure was repeated once again. After the beads were washed for the third time, the supernatant was discarded and 50 μl of 1 mM target solution in the selection buffer was added to them. The solution was pipetted few times up and down and incubated for 15 min at 21 °C and 900 rpm. Afterward, plate was moved to the magnet and 45 μl of the eluate was transferred to the 53 μl of RT‐PCR mix with the final concentration of: 1x GoTaq buffer, 1.35 mM Mg^2+^, 2 mM DTT, 1 μM forward primer, 1 μM reverse primer, and 0.3 mM dNTPs. The mix was first heated up to 65 °C for 5 min to break the secondary structure of the RNA. Then, 1 μl of M–MLV enzyme (Promega, USA) and 1 μl GoTaq polymerase (Promega, USA) were added to the mixture and RNA was reverse transcribed and amplified with the program: 1) 55 °C 10 min, 2) 95 °C 2 min; 3) 95 °C 1 min, 4) 56 °C 1 min, 5) 72 °C 1 min, and 6) 72 °C 3 min, with repeated 3‐5 steps. Afterward, 10 μl of the PCR product was transferred to the 38 μl of the transcription mix with final concentration: 40 mM Tris pH 7.9, 5 mM DTT, 2.5 mM NTPs, 25 mM Mg^2+^, 4 μM 12‐nt capture oligodeoxynucleotide (ODN), and the rest of the PCR product was stored at 4 °C. 1 μl of T7‐RNA polymerase (NEB, USA) was added to the transcription mixture and the in vitro transcription was performed for 2 h at 37 °C with gentle shaking. Afterward, half of the transcription mixture was discarded (25 μl) and 100 μl of 0.5 mg beads solution was added to it in final concentration of 1x PBS with 5 mM Mg^2+^. The mixture was incubated for 30 min to allow RNA‐ODN complexes and beads to interact. Finally, the beads were washed 3‐times with 100 μl of the selection buffer as done in the beginning of the round. Repeated rounds were done in the same way with increasing stringency by decreasing target concentration (1–0.05 mM), decreasing time of incubation of beads with the target solution (15–5 min), and decreasing the transcription time (2–1 h). The selection was done for 12 rounds.

##### Reverse Transcription and PCR Performance on Beads

45 μl of the eluate with dissociated RNA and target was transferred to the 54 μl of RT mix: 1x *M*‐MLV buffer, 0.5 μM biotinylated reverse primer, 0.5 mM dNTPs. Biotinylated reverse primer was purchased from Ella Biotech, Germany (5′‐biotin‐18spacer‐GTCCTGTGGCATCCACGAAA‐3′). First, the mixture without the *M*‐MLV enzyme (Promega, USA) was heated up to 65 °C for 5 min to break the secondary structure of the RNA. Afterward, enzyme was added and RT was performed for 15 min at 40 °C. Next, 0.25 mg Dynabeads *M*‐280 Streptavidin (ThermoFisher Scientific, USA) was added to the RT mix and was incubated for 30 min at 21 °C with gentle shaking. The supernatant was removed and a fresh PCR mix was added to the beads. PCR was performed for different number of PCR cycles with the program: 1) 95 °C 2 min; 2) 95 °C 1 min, 3) 56 °C 1 min, 4) 72 °C 1 min, and 5) 72 °C 3 min, with repeated 2−4 step. After the PCR, the solution was removed from the beads.

##### Radioactive Binding Studies

RNA was prepared as described in *Manual preparation of RNA‐oligo‐beads complexes for the initial round* in lower volume (300 μl) of PCR and transcription solution and was purified on a small 10% Urea‐PAGE gel. 75 pmol of pure RNA was used for 5′ end labeling of the RNA. First, RNA was dephosphorylated, 5′‐labeled with γ‐^32^ P‐ ATP (Perkin Elmer, USA), and purified with polyacrylamide gel electrophoresis. Next, one quarter of the RNA (app. 15 pmol) was mixed with 15 pmol of 12‐nt complementary oligo in the selection buffer (PBS + 3 mM Mg^2+^, pH 7.4). The solution was incubated for 1 h by heating up to 50 °C for 15 min and slowly cooling down to 30 °C with gentle shaking to allow RNA and oligos to hybridize. In the meantime, 0.5 mg of Dynabeads *M*‐280 Streptavidin was washed 3‐times with the selection buffer by using DynaMag‐2 Magnet and finally resuspended in 50 μl of the selection buffer. RNA‐oligos solution was then mixed with beads solution and incubated for 30 min at 21 °C with gentle shaking, so the biotinylated oligos and streptavidin coupled beads could interact. Afterward, the supernatant was removed and saved for measurement. Beads were washed 3‐times with 100 μl of selection buffer and each fraction was saved for the measurement. Finally, 50 μl of 1 mM target solution was added to the beads and was incubated for 15 min at 21 °C and 900 rpm. Eluate was removed from beads and both fractions were saved for measurement. 1 mL of ddH_2_O was added to each fraction and measured by liquid scintillation counter (Perkin Elmer, USA). Final result was calculated as a percentage of the sum of all fractions: supernatant, three washes, elution, and beads. Graph design and statistics were performed using GraphPad Prism 8 software.

##### NGS

The starting library, the selection round pool 2, 4, 6, 8, 10, and 11 or 12 were used for NGS. The protocol was taken from Tolle and Mayer.^[^ ^38^ ^]^ Pools were amplified with different primers synthetized by Ella Biotech, Germany, each having a unique 6‐nucleotides extension at the 5′ prime end, which was used as a barcode for further NGS analysis. A PCR reaction mix was prepared for each pool and for an additional No Template Control (NTC), using a proofreading *Pfu*‐Polymerase (homemade). 1 μM of reverse and 1 μM of forward primer with the same index sequence were added to the PCR master mix. The mix was split and the DNA template or water for NTC was added to it. DNA was amplified with a normal PCR program, described in *Manual preparation of RNA‐oligo‐beads complexes for the initial round*. PCR product was purified with Nucleospin Gel and PCR clean‐up columns (Macherey Nagel, Germany) according to the manufacturer's instructions. 0.125 μg of each DNA was mixed together and used for the following step. Next, adapter needed for immobilization and processing of the sample on the Illumina instruments was ligated to the DNA sequences using TruSeq DNA PCR‐Free Sample Preparation Kit LT (Illumina, USA). Ligation was done according to manufacturer instructions applying three procedures: “End Repair”, “Adenylation”, and “Adapter Ligation”. The ligated products were afterward separated on an agarose gel, cutting out only the longest sequence (app. 210 bp) with ligated adapters on both sides. The DNA was purified from the gel using Nucleospin gel and PCR clean‐up columns, following manufacturer instructions. Finally, DNA was eluted with the resuspension buffer (TruSeq DNA PCR‐Free Sample Preparation Kit LT) and was used for illumina sequencing with NextSeq500 with High Output v2 chemistry. NGS analysis was done by in‐house designed software AptaNext. Graph design was performed using GraphPad Prism 8 software.

##### RNA Displacement from Complementary Fluorescence Labeled Oligodeoxynucleotides by Target Addition Measured by Fluorescent Polarization

5′‐Cy3 labeled oligodeoxynucleotides (ODN) were purchased from Ella Biotech, Germany. Sequences of different oligo lengths are provided in Table 2, Supporting Information. Polarization assay was performed in final volume of 50 μl of (100 nM Cy3‐ODN, 500 nM RNA, 0.1−1 mM target) in 1xPBS with 3 mM Mg^2+^ and 0.005% Tween 20. First, RNA and target were mixed together and incubated for 30 min at 21 °C and 500 rpm. Second, Cy3‐ODN was added and incubated for another 30 min at 21 °C and 500 rpm. Finally, the solution was transferred to the Corning 96 Well Half‐Area Microplate (Sigma Aldrich, Germany) and was measured by *Tecan Ultra* Micro Plate Reader (Tecan, Switzerland) at excitation light 535 nm, and emission light 595 nm. The fluorescence polarization (FP) values were always normalized to the value of the 100 nM Cy3‐ODN sample. Graph design was performed using GraphPad Prism 8 software.

##### ITC Measurement

The RNA for affinity measurement was prepared by in vitro transcription. After ethanol precipitation, the RNA was resuspended in the binding buffer and further washed several times with the same buffer on the 3 K Amicon Ultra 0.5 mL Centrifugal Filters (Merck, Germany). Different concentrations of ligands in the syringe were titrated to different aptamer concentrations in the sample cell, dependently of the ligand‐aptamer pair. For the titrations with neomycin B‐ and kanamycin‐aptamers pairs, we titrated the ligand in 38 injections: first injection of 0.4 μl and subsequent 37 injections of 1 μl. With theophylline‐ and riboflavin‐aptamer pairs, we titrated the ligand in 38 injections 0.4 μl and subsequent 18 injections of 2 μl. The affinity was measured on MicroCalTM PEAQ‐ITC (Malvern Panalytical, UK) with power of 10 μcal/sec and spacing time 180 sec. Graph design was performed using GraphPad Prism 8 software.

## Conflict of Interest

The authors declare no conflict of interest.

## Supporting information

Supplementary Material

## Data Availability

The data that support the findings of this study are available from the corresponding author upon reasonable request.
