# Supplementary material for: Robotic‐Assisted Capture‐Systematic Evolution of Ligands by Exponential Enrichment of RNA Aptamers Binding to Small Molecules
Source: Chembiochem. 2025 Jul 9;26(15):e202500264. doi: 10.1002/cbic.202500264 (PMC12376241; doi:10.1002/cbic.202500264)
Supplement: Supplementary file 1 — Supplementary Material [file CBIC-26-e202500264-s001.pdf]

Supplementary information

Robotic-assisted selection of RNA aptamers binding to small molecules

Tjasa Legen[1,2], Günter Mayer[1,2]*

[1]Life and Medical Sciences Institute (LIMES), University of Bonn, Germany
[2]Center of Aptamer Research and Development (CARD), University of Bonn, Germany

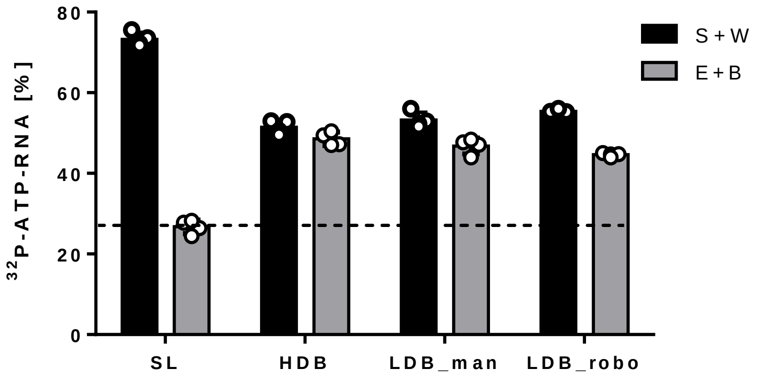

Figure S1. Annealing efficiency of the starting library (SL) and enriched pools selected for neomycin B by different strategies. During the selection, annealing efficiency of enriched sequences increases. Error bars show standard deviation (n=2). S+W: Sum of supernatant and three washing fractions, E+B: Sum of elution and beads fraction. HDB: selection with high-density beads, LDB: selection with low-density beads, man: manual selection, robo: robotic selection.

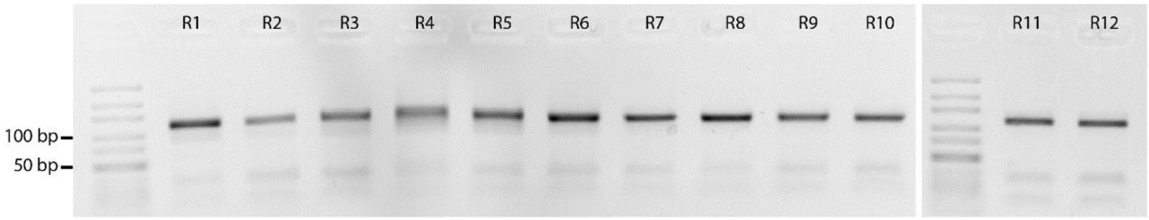

Figure S2. PCR profile of different selection rounds of robotic assisted selection targeting neomycin B. PCR product was loaded on a 4% Agarose gel with Ultra-low range ladder.

Table S1. Summary of SELEX conditions utilized in the selections targeting neomycin B

<table>
<tr><th>SELEX round</th><th>Target concentration</th><th>IVT time</th><th>Washing</th><th>Incubation time</th></tr>
<tr><td>1</td><td rowspan="4">1 mM</td><td>50°C-30°C</td><td>1x100 µl: 5 min; 2x100 µl</td><td rowspan="4">15 min</td></tr>
<tr><td>2</td><td rowspan="2">2 h</td><td rowspan="11">3x100 µl</td></tr>
<tr><td>3</td></tr>
<tr><td>4</td><td rowspan="4">1.5 h</td></tr>
<tr><td>5</td><td rowspan="4">0.5 mM</td><td rowspan="4">10 min</td></tr>
<tr><td>6</td></tr>
<tr><td>7</td></tr>
<tr><td>8</td><td rowspan="5">1 h</td></tr>
<tr><td>9</td><td rowspan="4">50 µM</td><td rowspan="4">5 min</td></tr>
<tr><td>10</td></tr>
<tr><td>11</td></tr>
<tr><td>12</td></tr>
</table>

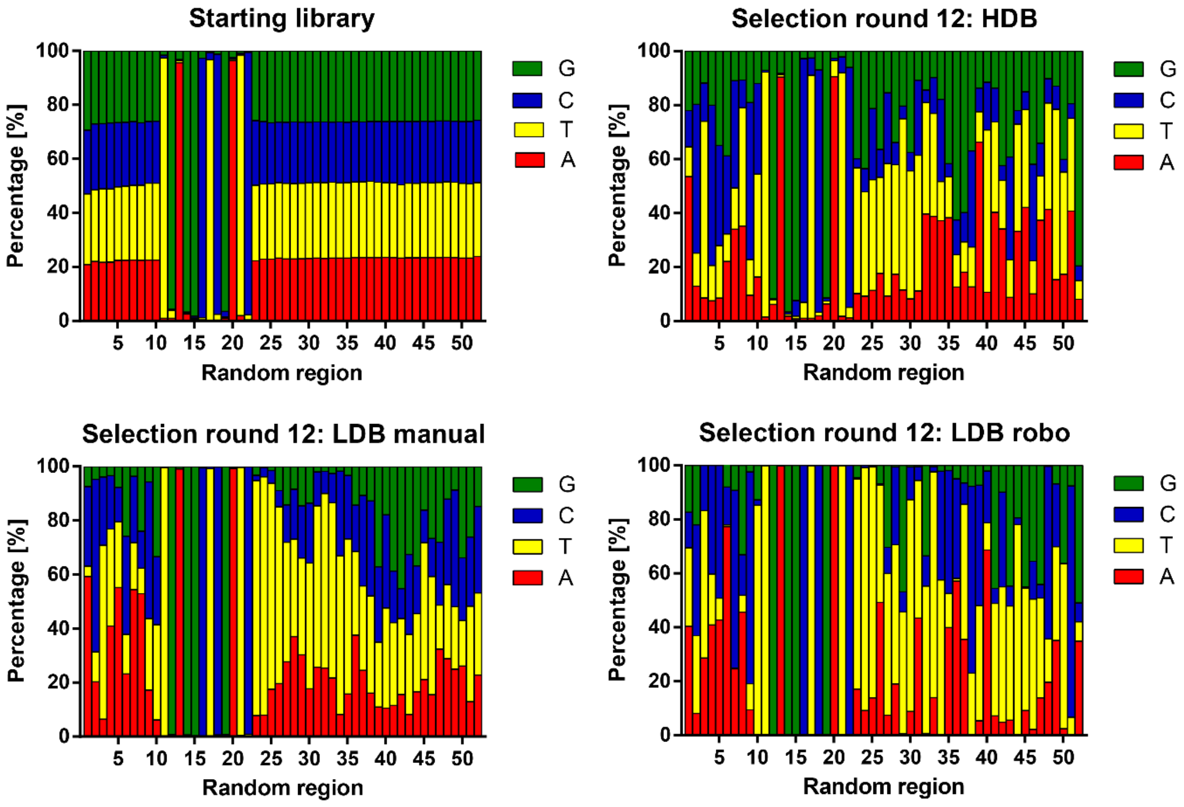

Figure S3. Nucleotide distribution in the random region of starting library, round 12 (R12) high-density beads (HDB) selection, R12 low-density beads (LDB) manual selection, and R12 LDB robotic selection targeting neomycin B measured by next-generation sequencing (NGS).

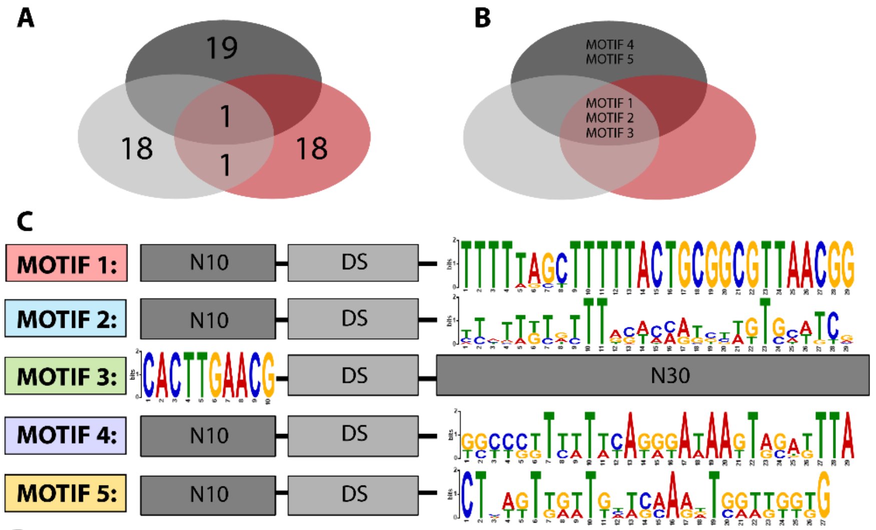

D

HDB_1	ACCCCATCT	**TGAGGCTCGATC**	GGCTCGTTTTTCAGGGATAAGTAGATTTAG
HDB_2	ACCGGCACC	**TGAGGCTCGATC**	TTTGTTTGCAAAGGGCATTGCATCGATGTG
HDB_3	ACTCCCATCT	**TGAGGCTCGATC**	GGCTCGTTTTTCAGGGATAAGTAGATTTAG
HDB_4	ACCGGCACC	**TGAGGCTCGATC**	TTTGTTTGCAAAGGGCATTGCATCGATGTG
HDB_5	GGGTACTGA	**TGAGGCTCGATC**	TGGATAGTTACGGCACACCTCGACCAAAAG
HDB_6	GGTGTACTGA	**TGAGGCTCGATC**	TGGATAGTTACGGCACACCTCGACCAAAAG
HDB_7	ATTCACTCC	**TGAGGCTCGATC**	TGGCTCAGTGAATGCACCAGTTGGTTTGGG
HDB_8	TATTCACTCC	**TGAGGCTCGATC**	TGGCTCAGTGAATGCACCAGTTGGTTTGGG
HDB_9	ACCCCATCT	**TGAGGCTCGATC**	GGCTCGTTTTTTCAGGGATAAGTAGATTTAG
HDB_10	ACTCCCATCT	**TGAGGCTCGATC**	GGCTCGTTTTTTCAGGGATAAGTAGATTTAG
HDB_11	CACGAACG	**TGAGGCTCGATC**	TTTTTAGCTTTTTACTGCGGCGTTAACGGT
HDB_12	CACTTGAACG	**TGAGGCTCGATC**	TTTTTAGCTTTTTACTGCGGCGTTAACGGT
HDB_13	ACCCGTAAC	**TGAGGCTCGATC**	GCAATTTTATATATATGAAGGGCATCGTAGT
HDB_14	ACTCCGTAAC	**TGAGGCTCGATC**	GCAATTTTATATATATGAAGGGCATCGTAG
HDB_15	ACCCGGCCC	**TGAGGCTCGATC**	TTTGTTTGCAAAGGGCATTGCATCGATGTGT
HDB_16	ACCCGGCTCC	**TGAGGCTCGATC**	TTTGTTTGCAAAGGGCATTGCATCGATGTG
HDB_17	CGCTGTACT	**TGAGGCTCGATT**	GTTGTTTTTGAAAATGATGGTTGGTCCATAT
HDB_18	CTGCTGTACT	**TGAGGCTCGATT**	GTTGTTTTTGAAAATGATGGTTGGTCCATA
HDB_19	GCACCT	**TGAGGCTCGATC**	ATTTGTTGATCAAAGTCGATGGTGACTGGGTT
HDB_20	ACTCTGGCAT	**TGAGGCTCGATC**	GTTTAGTTGTTTTTATTGTTGAGTTCCTGC

LM_1	CACTTGAACG	**TGAGGCTCGATC**	TTTTTAGCTTTTTACTGCGGCGTTAACGGT
LM_2	ACTAACAGTC	**TGAGGCTCGATC**	TTTTGCAGAAACTTTCGTCTTTTGGCTATC
LM_3	CACTTGAACG	**TGAGGCTCGATC**	TTTTTAGCTTTTTTACTGCGGCGTTAACGGT
LM_4	ACTTAATTCG	**TGAGGCTCGATC**	TTAGCTTTTTTCAGTCACTAGCATCTAGTA
LM_5	ACTAACAGAC	**TGAGGCTCGATC**	TTTTTTAACAACCAAACATGTGTCGTCCAC
LM_6	ACTAAGCACT	**TGAGGCTCGATC**	TTTTACACTTTTTGGCCTAGCATTCATCTT
LM_7	ACTAAACACT	**TGAGGCTCGATC**	TTTATTGTATTTACACCATCTTGTGCATCA
LM_8	ACTACGCACT	**TGAGGCTCGATC**	TTTAAAACTTTTTTCACTGGGCTAACACCT
LM_9	ACTAACAGTC	**TGAGGCTCGATC**	TTTTAATGATTTTTCACTGTGTCGGTCATC
LM_10	GTCCAACACT	**TGAGGCTCGATC**	AAATAACTTTAACCACTGAGCATGCCACCA
LM_11	ACTTGCACTT	**TGAGGCTCGATC**	TTTTCAATTTCACTGGGTATTCAGAAGTCG
LM_12	CTTCATCAAA	**TGAGGCTCGATC**	TTTTTATTTAACCATCTGTAAGCATTTACG
LM_13	ACTCATTATT	**TGAGGCTCGATC**	TTTATCAGTTTTTCACCTGAGTATTCATGC
LM_14	ACTAACCACT	**TGAGGCTCGATC**	TTTTTATCACTTTTAGAGTCTGAACGCATC
LM_15	CTTCAGTGCT	**TGAGGCTCGATC**	TTTTGTTCCAGTTTATGGCGTAAGCGATTG
LM_16	GCTAACACTC	**TGAGGCTCGATC**	TTTTCATATTCTTTTACCTGGCGCATCACA
LM_17	ACTAGTAACT	**TGAGGCTCGATC**	TTACTTTATACTCAATGTGGCTTTGCAACT
LM_18	GAATCCAG	**TGAGGCTCGATC**	TTTTTAATTTTTCATTCCTAGCATCGCGTT
LM_19	CTTGAACACT	**TGAGGCTCGATC**	TTTTGCTTAATTCACCTTGTCATTCTCGAA
LM_20	CATAACA	**TGAGGCTCGATC**	TTTACTATTCTTTTATCCGTGTCAGCACAC

LR_1	ACTAAACACT	**TGAGGCTCGATC**	TTTATTGTATTTACACCATCTTGTGCATCA
LR_2	TTACCACGCT	**TGAGGCTCGATC**	TTTTGCTTTGTCCATCTAGTGTTGTCTGCG
LR_3	GGTTCCGCAT	**TGAGGCTCGATC**	AAAACTTTTCATCATTCTGTGTACGTCTCG
LR_4	GGCCAAATCT	**TGAGGCTCGATC**	ATTGAACAATTCCATGTCATTGTGTAGTCC
LR_5	CACTTGAACG	**TGAGGCTCGATC**	TTTTTAGCTTTTTACTGCGGCGTTAACGGT
LR_6	CGTCCAACTG	**TGAGGCTCGATC**	TTAATTTTCGACTAGTTCCAAGGCATCTTG
LR_7	ACTACCAGTT	**TGAGGCTCGATC**	GTTTTAGCTTTTAGTCAATTGTGTCATTCG
LR_8	GGCTCACAGC	**TGAGGCTCGATC**	TTTTGTTAACCGGCGTTGTGGCTAATCATG
LR_9	TCCACTACGT	**TGAGGCTCGATC**	TACTTTTTTCGTTTCGTGGATGC
LR_10	GGCCAAACTT	**TGAGGCTCGATC**	ATTCATCATTTTCATGCCAGTGGATATACG
LR_11	CACTTGAACG	**TGAGGCTCGATC**	TTTTTTAGCTTTTTACTGCGGCGTTAACGG
LR_12	CACTTGAACG	**TGAGGCTCGATC**	TTTTTAGCTTTTAGTCAATTGTGTCATTCG
LR_13	CTTCACACAC	**TGAGGCTCGATC**	ACCTTTAGTCTTTTACTTGTAGCTTGCACG
LR_14	ACTAAACACT	**TGAGGCTCGATC**	TTTATTGTATTTACACCATCTTGTGCATCG
LR_15	CACTTGAACG	**TGAGGCTCGATC**	TTTTTAGCTTTTTACTGCGGCGTTAACGG
LR_16	ACTAAACACT	**TGAGGCTCGATC**	TTTTGCTTTGTCCATCTAGTGTTGTCTGCG
LR_17	TTACCACGCT	**TGAGGCTCGATC**	TTTATTGTATTTACACCATCTTGTGCATCA
LR_18	CACTTGAACG	**TGAGGCTCGATC**	TTTTTAGCTTTTTACTGCGGCGTTAACGGC
LR_19	CACTTGAACG	**TGAGGCTCGATC**	GTTTTAGCTTTTAGTCAATTGTGTCATTCG
LR_20	CACTTGAACG	**TGAGGCTCGATC**	TTTTTAGCTTTTTACTGCGGCGTTAACGGG

Figure S4. The sequence analysis of high-density beads (HDB) selection, low-density beads (LDB) manual selection, and LDB robotic selection for neomycin B. A) Common sequence in different selection strategies among 20 the most enriched sequences of each selection. Dark grey: HDB selection; light grey: LDB manual selection; red: LDB robotic selection. **B)** Common motifs in different selection strategies among 20 the most enriched sequences of each selection. For motifs search, MEME suite tool for motif discovery was utilized.[1] **C)** Motif sequence and positions of the motifs in the enriched sequences. **D)** Highlighted motif sequences in the 20 the most enriched sequences in each neomycin selection (HDB: High-density beads, LM: Low-density beads manual, LR: Low-density beads robotic). With the black arrow, the sequences found among all three selections are marked and with the red arrow, the sequence common in LM and LR selection.

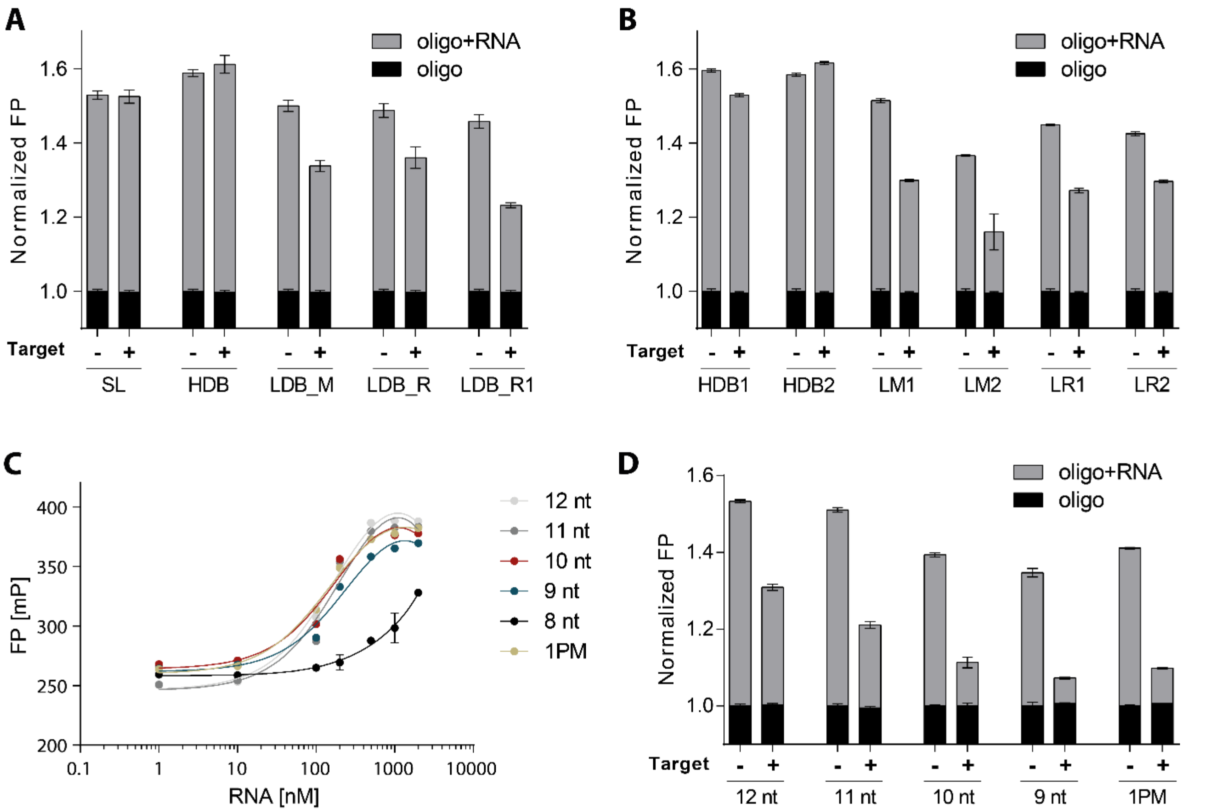

Figure S5. Optimization of fluorescence polarization assay for binding studies using enriched pool and monoclonal sequences binding to neomycin B. A) Binding of enriched pools to neomycin target obtained by different selections measured by fluorescence polarization assay. For assay, 100 nM of Cy3 labelled capture oligodeoxynucleotide (ODN) and 500 nM RNA was used in the absence or presence of 100 µM neomycin. The FP-values were normalized according to the value of free Cy3-ODN. Error bars show standard deviation (n=3). SL: starting library, HDB: Selection with high-density beads, LDB_M: Manual selection with low-density beads, LDB_R: Robotic selection with low-density beads, LDB_R1: Robotic selection with low-density beads and RT-PCR performed on magnetic beads (see **Supp. Fig. 9**). **B)** Binding of different monoclonal sequences to neomycin B target obtained by different selections measured by fluorescence polarization assay. For assay, 100 nM of Cy3 labelled capture ODN and 500 nM RNA was used in the absence or presence of 100 µM Neomycin. The FP-values were normalized according to the value of free Cy3-ODN. Error bars show standard deviation (n=3). HDB: Sequences obtained by high-density selection, LM: Sequences obtained by manual low-density selection, LR: Sequences obtained by robotic low-density selection. **C)** Annealing efficiency of RNA to different length ODNs measured by fluorescent polarization. For the annealing, different concentration of neomycin B aptamer LM1 were used during 30 min incubation at 21°C in PBS with 3 mM Mg^{2+}. Concentration of the ODNs was constant 100 nM. The FP-values were normalized according to the value of free Cy3-ODN. Error bars show standard deviation (n=3). The data were fitted in non-linear fit model. By the 8-nt capture ODN, there is only a weak annealing observed. nt: nucleotide, 1PM: one point mutation in 12-nt oligonucleotide. **D)** LM1 aptamer displacement from different lengths of ODNs by binding to the neomycin B target. The concentration of ODN and aptamer LM1 was 100 nM and 500 nM, respectively. Concentration of neomycin was 100 µM. The FP-values were normalized according to the value of free Cy3-ODN. Error bars show standard deviation (n=3). The highest decrease in the fold-change of FP is by the 9-nt ODN.

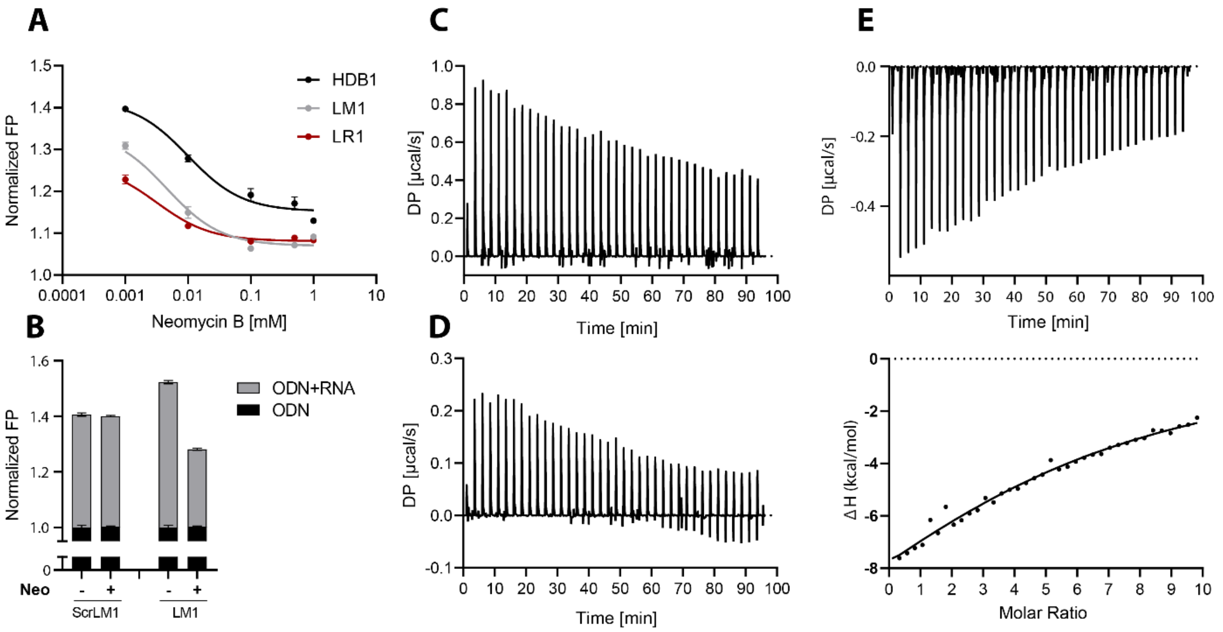

Figure S6. Binding affinity measurement by isothermal titration calorimetry (ITC). A) Concentration dependent binding of neomycin B to HDB1, LM1 and LR1 aptamer measured by fluorescence polarization. The FP-values were normalized according to the value of free Cy3-ODN. Error bars show standard deviation (n=3). The estimated K_d determined by the assay was approximately 10 µM, 4 µM and 3 µM, respectively. The fluorescence change was fitted to inhibitor vs. response model. FP: fluorescence polarization. **B)** Binding of scrabbled LM1 (ScrLM1) and LM1 sequences to neomycin B measured by fluorescence polarization assay. The FP-values were normalized according to the value of free Cy3-ODN. Error bars show standard deviation (n=3). **C)** Neomycin B (1 mM) titration to PBS + 3 mM Mg^{2+} buffer. DP: differential power. **D)** Kanamycin (1 mM) titration to PBS + 3 mM Mg^{2+} buffer. **E)** LR1 and kanamycin interaction. K_d is 130.0 ± 30.9 µM. For the measurement 20 µM of RNA and 1 mM of target was used.

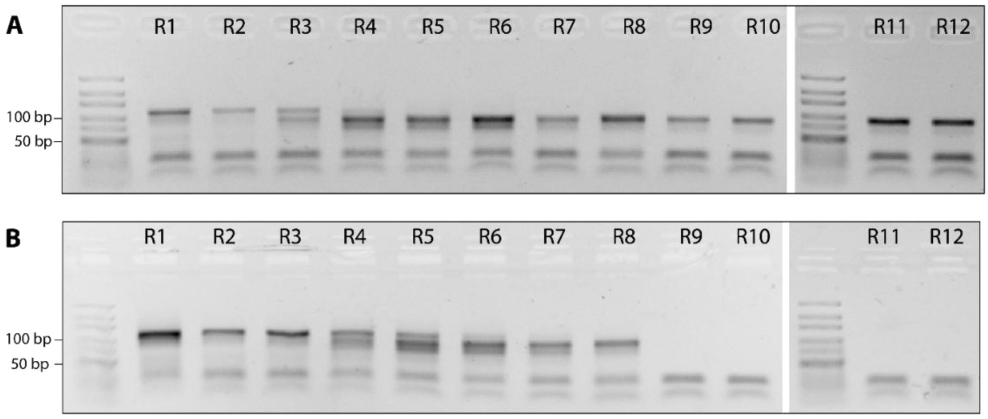

Figure S7. PCR profile of different rounds of robotic assisted selection targeting L-arginine (A) and theophylline (B). Both selections lead to truncation of the RNA library or even to loss of the product band.

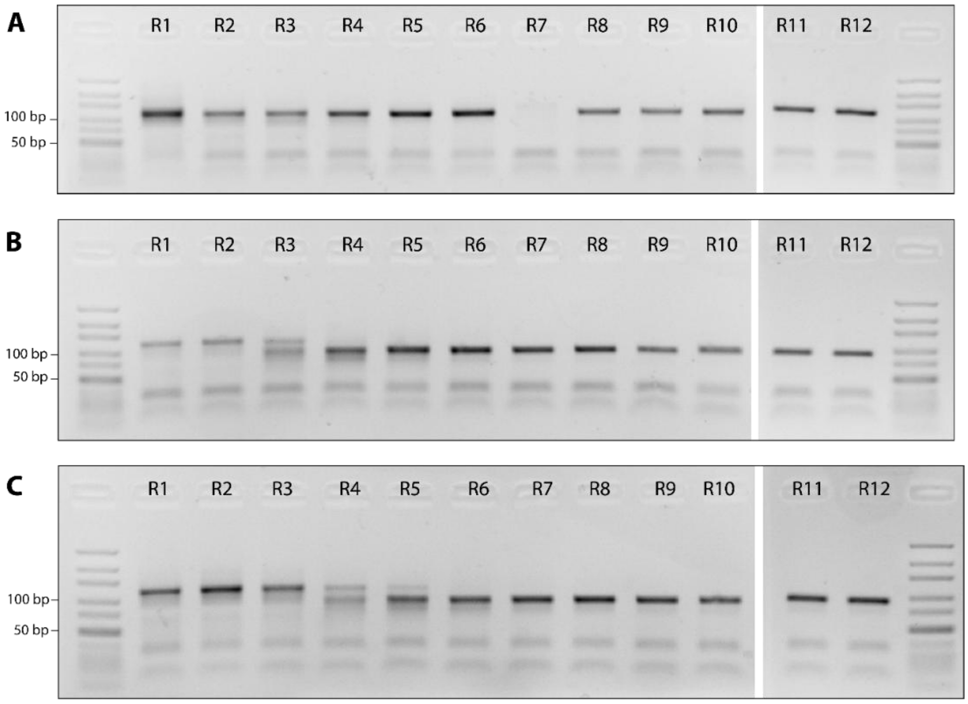

Figure S8. PCR profile of different rounds of automated selection targeting A) neomycin B with initial concentration of 50 µM, B) neomycin B with initial concentration of 5 µM, and C) only selection buffer (PBS + 3 mM Mg^{2+}). By initial target concentration as low as 5 µM (B), the library length shortens indicating unsuccessful selection, the same being observed in the selection using no target molecule (C). PCR product was loaded on a 4% Agarose gel with Ultra-low range ladder.

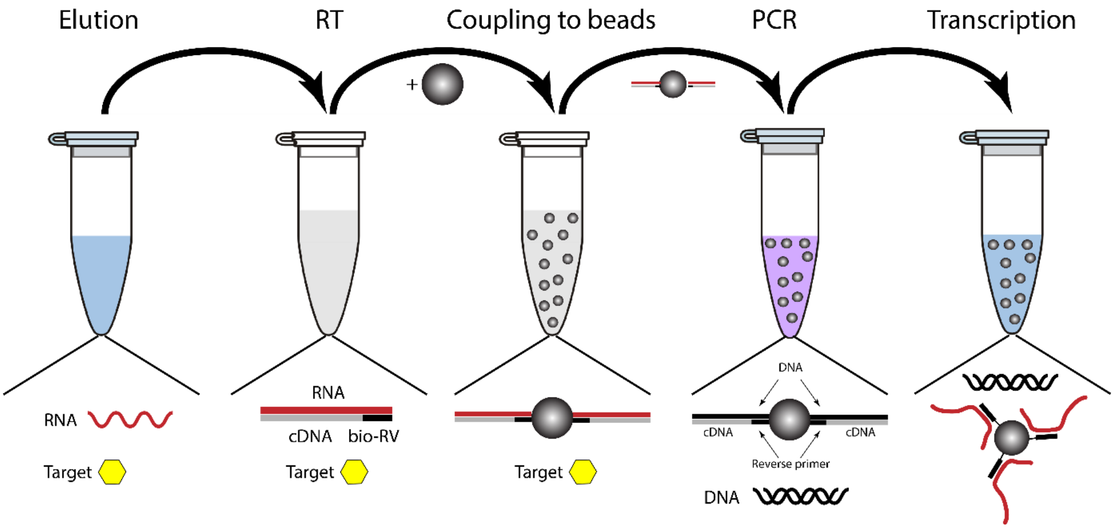

Figure S9. Separation of reverse transcription (RT) and amplification step of dissociated sequences during capture SELEX process to remove remaining target molecule. Elution mix with dissociated RNA and target molecule is mixed with the reverse transcription (RT) solution, which contains biotinylated reverse primer. Generated biotinylated-cDNA (bio-cDNA) is then captured by addition of streptavidin (SA) beads to the RT solution to remove the solution with the target molecule. Next, polymerase chain reaction (PCR) mix is added to the beads with captured cDNA template. After amplification, an aliquot of PCR product is added as a template for the *in vitro* transcription (IVT) reaction, where the transcribed RNA is directly annealed to the biotinylated-capture oligodeoxynucleotide present in the IVT mixture and subsequently captured to SA-beads.

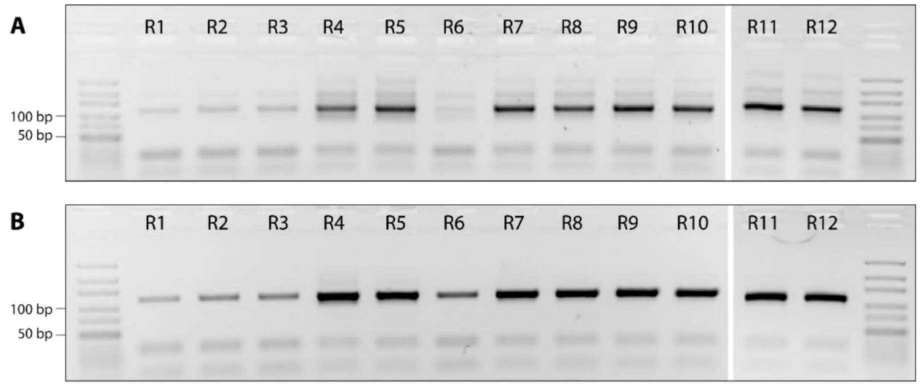

Figure S10. PCR profile of automated selection with a new strategy keeping RT and PCR reaction separated for removal of target molecule (A) and re-amplification of PCR product (B). All additional bands visible in the gel A disappear after the re-amplification (gel B) indicating that those bands were either RNA or single-stranded DNA sequences. The utilized target in the SELEX was neomycin B. PCR product was loaded on a 4% Agarose gel with Ultra-low range ladder.

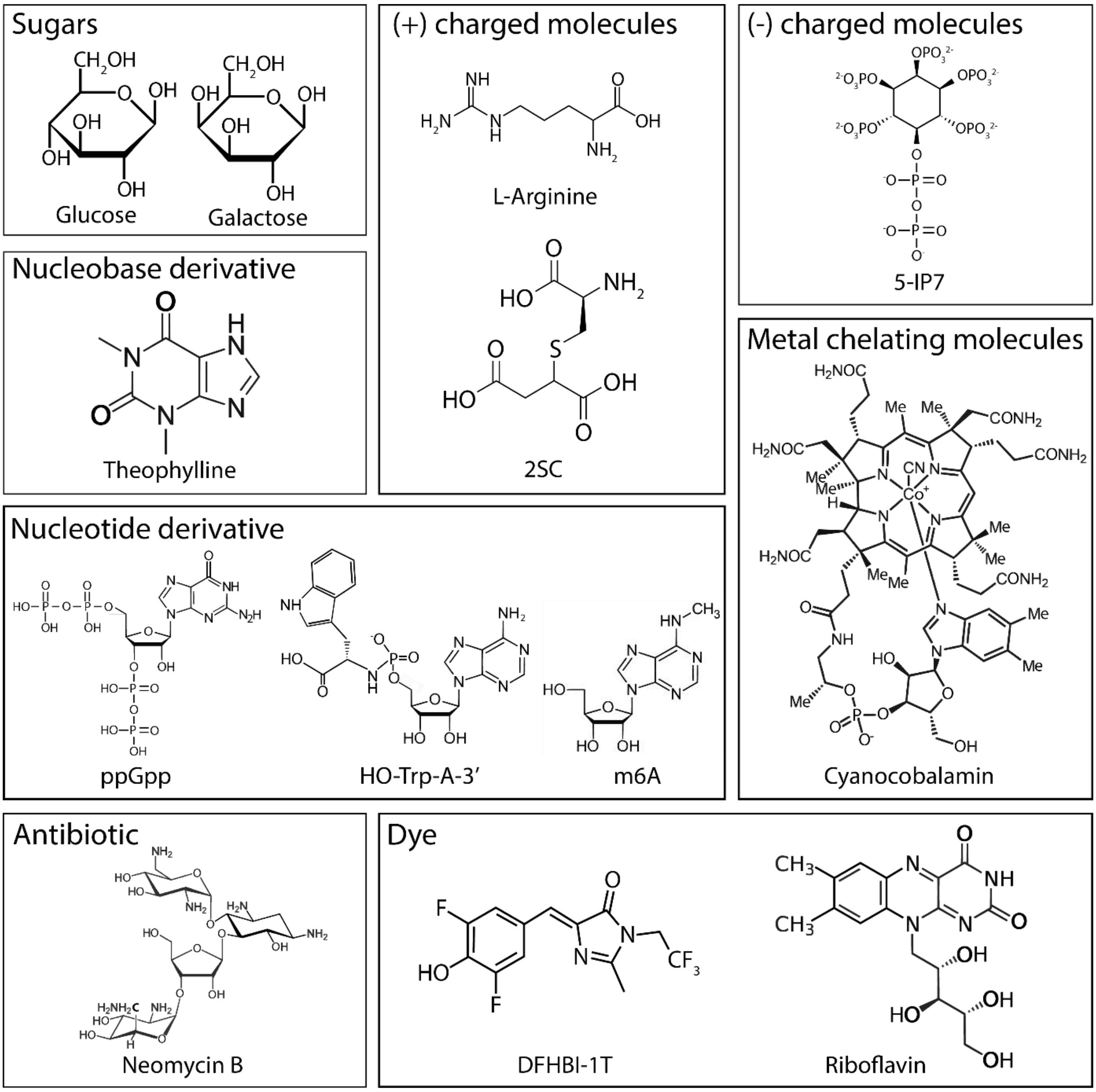

Figure S11. Structure and characterization of small molecule targets according to their functionality or functional groups. Different structural or functional moieties were chosen for the automated selection to investigate versatility of selection ability. 2SC: S-(2-succinyl) cysteine, DFHBI-1T: 3,5-Difluoro-4-hydroxybenzylidene imidazolinone-1T,[2] HO-Trp-A-3’: D-Tryptophanyl Adenosine 5'-Monophosphate Phosphoramidate,[3] m6A: N6-Methyladenosine, 5-IP7: 5-diphospho-*myo*-inositol pentakisphosphate,[4] ppGpp: Guanosine pentaphosphate.

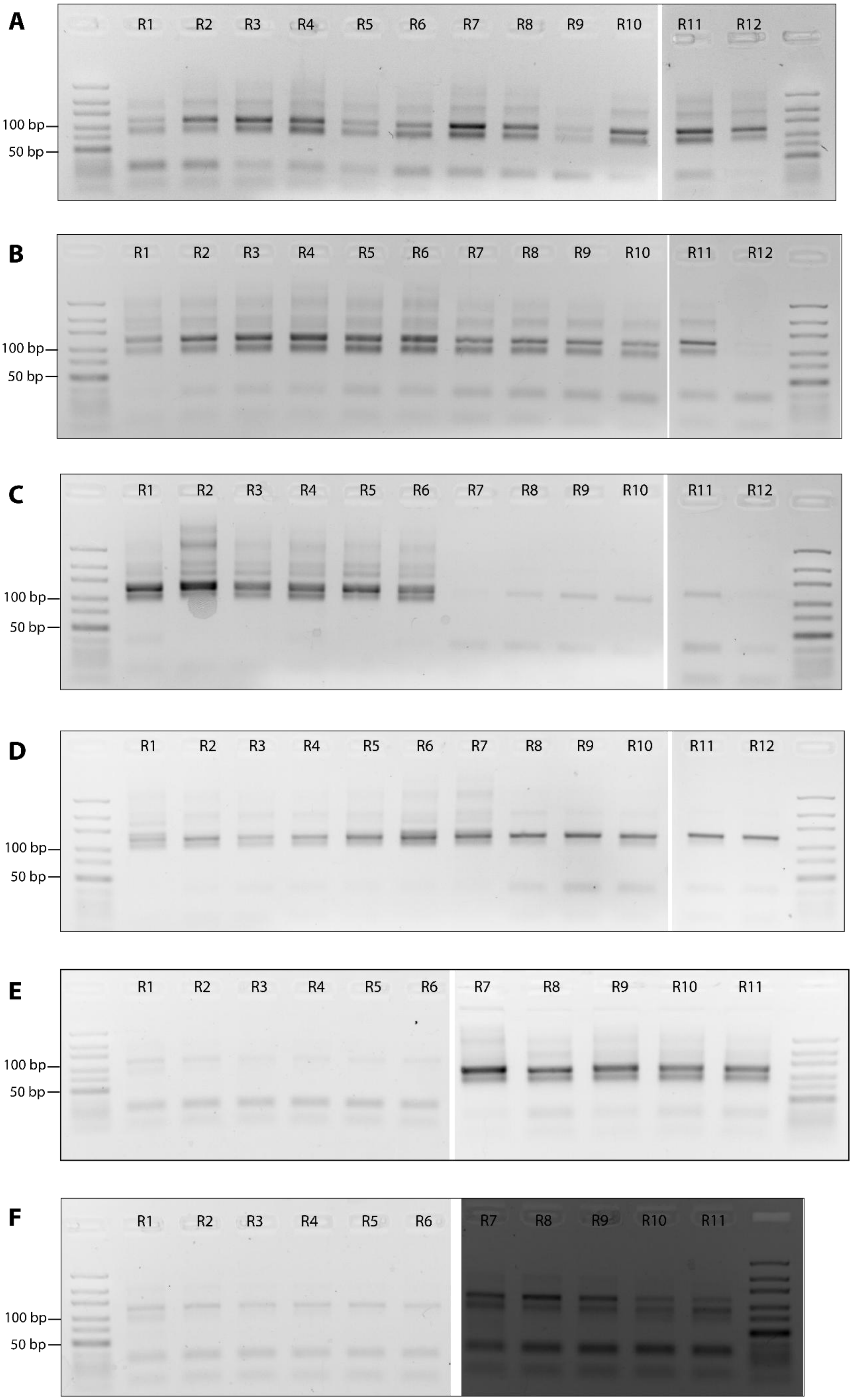
A
R1 R2 R3 R4 R5 R6 R7 R8 R9 R10 R11 R12
100 bp
50 bp
B
R1 R2 R3 R4 R5 R6 R7 R8 R9 R10 R11 R12
100 bp
50 bp
C
R1 R2 R3 R4 R5 R6 R7 R8 R9 R10 R11 R12
100 bp
50 bp
D
R1 R2 R3 R4 R5 R6 R7 R8 R9 R10 R11 R12
100 bp
50 bp
E
R1 R2 R3 R4 R5 R6 R7 R8 R9 R10 R11
100 bp
50 bp
F
R1 R2 R3 R4 R5 R6 R7 R8 R9 R10 R11
100 bp
50 bp

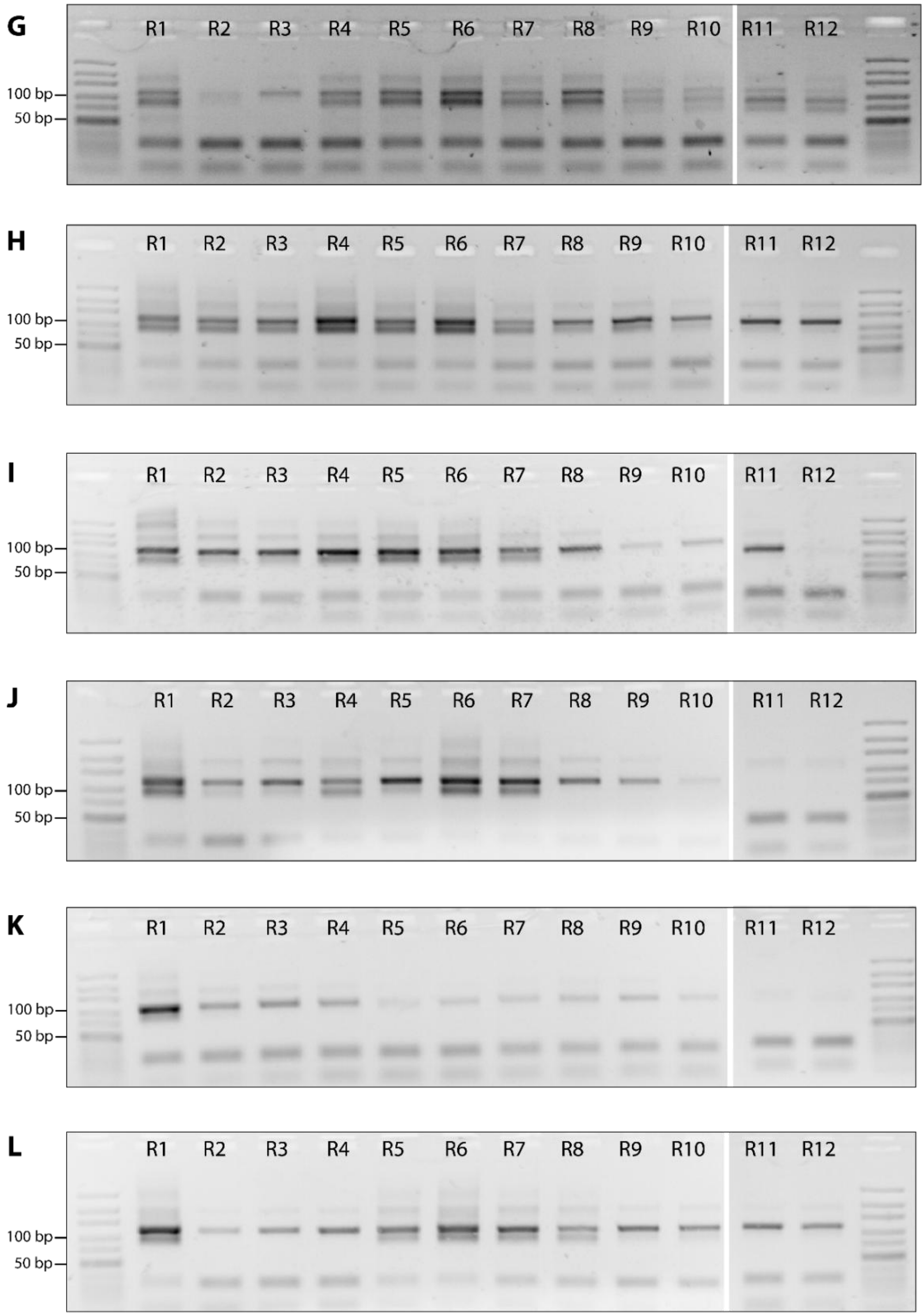

Figure S12. PCR profile of automated selection with various small molecule targets. A) Arginine, **B)** Theophylline, **C)** Galactose, **D)** 3,5-Difluoro-4-hydroxybenzylidene imidazolinone-1T (DFHBI-1T),[2] **E)** D-Tryptophanyl Adenosine 5'-Monophosphate Phosphoramidate (HO-Trp-A-3'),[3] **F)** N6-Methyladenosine (m6A), **G)** Guanosine pentaphosphate (ppGpp), **H)** 5-diphospho-*myo*-inositol pentakisphosphate (5-IP7),[4] **I)** Glucose, J) S-(2-succinyl) cysteine (2SC), **K)** Riboflavin, and **L)** Cyanocobalamin.

No.	Sequence	Arginine	Theo	DFHBI-1T	2SC	Riboflav	Cyanoco	Glucose	HO-Trp	m6A	5-IP7	ppGpp
1	GGAACGGTAATGAGGCTCGATCGAATTAGACCGCCTTGATTCCCTCGTGGCG	0	0	0.0869	0.2749	0.0042	0.2666	0.0405	0.0202	0.3329	9.1016	48.9888
2	CTATCGCTTGTGAGGCTCGATCTTTATGTGCCCCTCCTTCAGGTTGGCGTCT	0	0	0	0	0	0	0	0.0554	0.1967	0	0
3	GGGATGTCGTGAGGCTCGATCAATGTATTTTGCTCCCTGCTCGGTGGTTCC	0	0	0	0.2227	0.0021	0.034	0.3061	0	0.3138	0	0.0106
4	CCACGCTGGTGAGGCTCGATCACGGAGACTTCATTTCCCATTGTTGTGTGG	0.2252	0	0	0	0	0	0	0.586	1.8388	0.0124	0.907
5	GCTATCTAGTTGAGGCTCGATCAGTGTACCATACTTTCTTCTCCCACCGGCA	0	0	0.0228	0.0224	0	0.0059	0.0979	0	0.0536	0	0
6	ATCTATCCGATGAGGCTCGATCCAGTGCTTTTGCTCTCTAGAGTCATTCACG	0	0	0.2513	0.0077	0	0.8026	0.0586	0	0	0	0
7	CCGGTATCTATGAGGCTCGATCTTCTTACTACCTTCTCTTCAGGTCGGCGCCG	0	0	0	0	0	0.0221	0.0052	1.108	0.7844	0	0.0301

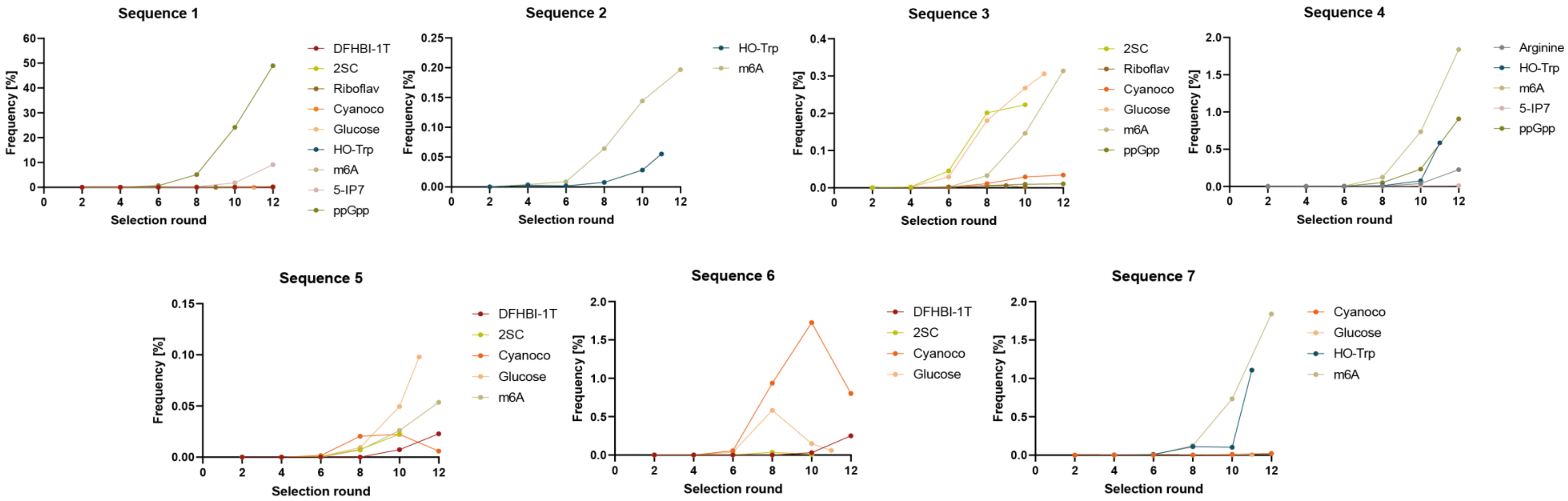

Figure S13. Enrichment of parasitic non-binding sequences in different selections. In the table, seven sequences are listed, which were found among several selections targeting different molecules. The enrichment of each sequence in different selection is shown in the graphs below. DFHBI-1T: 3,5-Difluoro-4-hydroxybenzylidene imidazolinone-1T,[2] Theo: Theophylline, 2SC: S-(2-succinyl) cysteine, Riboflav: Riboflavin, Cyanoco: Cyanocobalamin, HO-Trp: D-Tryptophanyl Adenosine 5'-Monophosphate Phosphoramidate,[3] m6A: N6-Methyladenosine, 5-IP7: 5-diphospho-*myo*-inositol pentakisphosphate,[4] ppGpp: Guanosine pentaphosphate.

	Neomycin B			Frequency [%]						
		FAMILY	SEQUENCE	Round 2	Round 4	Round 6	Round 8	Round 10	Round 12	PM
LM1 →	**N1**	Neo1	CACTTGAACGTGAGGCTCGATCTTTTTAGCTTTTTTACTGCGGCGTTAACGGT	0.0204	3.4954	12.0294	13.8122	20.7139	23.1198	0
	N9	Neo1	CACTTGAACGTGAGGCTCGATCTTTTTAGCTTTTTTTACTGCGGCGTTAACGG	0.0004	0.1079	0.3815	0.4596	0.9392	1.3032	2
	N10	Neo1	CACTTGAACGTGAGGCTCGATCTTTTTAGCTTTTTACTGCGGCGTTAACGGTT	0.066	9.7189	20.4801	14.2551	4.0027	1.0151	2
	...	Neo1								
	N2	Neo2	CCACTACGTTGAGGCTCGATCTTTTGCTTTGTCCATCTAGTGTTGTCTGCGT	0.0001	0.0778	0.4855	1.5797	6.6098	19.6315	0
	N3	Neo2	ACCACGCTTGAGGCTCGATCTTTTGCTTTGTCCATCTAGTGTTGTCTGCGT	0.0222	6.9117	16.6203	24.6762	23.6135	13.4118	5
	N6	Neo2	CACCACGCTTGAGGCTCGATCTTTTGCTTTGTCCATCTAGTGTTGTCTGCGT	0.0001	0.0595	0.2981	0.661	1.9265	4.1765	3
	...	Neo2								

	Arginine			Frequency [%]						
	ID	FAMILY	SEQUENCE	Round 2	Round 4	Round 6	Round 8	Round 10	Round 12	PM
	**A1**	Arg1	CCACTTCGGATGAGGCTCGATCAACTCCCGTTATAGTCTGGGCAATCGCTGG	0	0	0.0006	0.0155	0.1648	1.4959	0
4 →	A2	Arg2	CCACGCTGGTGAGGCTCGATCACGGAGACTTCATTTCCCATTGTTGTGTGGT	0	0	0.0004	0.0044	0.0391	0.2252	0
	A3	Arg3	GCCCCACGTCTGAGGCTCGATCTACTTGAGTTCATTCTTCATGCATCCCGCG	0	0	0.0001	0.0003	0.0182	0.1678	0
	A4	Arg4	GACCTGCTCGTGAGGCTCGATCCTCCTAATTAGTGCCGTCGGTCGTCCCGCG	0	0	0.0002	0.0031	0.0277	0.1656	0
	A5	Arg5	CCATGCCCCGTGAGGCTCGATCACTCCCAGACCTGTAGTTCGCGTCCCGTGC	0	0.0001	0.0013	0.0082	0.0591	0.136	0

Theophylline			Frequency [%]						
ID	FAMILY	SEQUENCE	Round 2	Round 4	Round 6	Round 8	Round 10	Round 11	PM
**T1**	Theo1	CTATCGCTTGTGAGGCTCGATCTTTATGTGCCCCTCCTTCAGGTTTGGCGTCT	0	0.0002	0.0272	1.1351	7.6954	10.6657	0
T12	Theo1	CTATCGCTTGTGAGGCTCGATCTTTATGTGCCCCTCCTTCAGGTTTGGCGCCT	0	0	0.0001	0.0133	0.121	0.2351	1
T29	Theo1	CTATCGCTTGTGAGGCTCGATCTTTATGTGCCCCTCCTTCAGGCTTGGCGTCT	0	0	0	0.0052	0.0478	0.1018	1
T44	Theo1	CTATCGCTTGTGAGGCTCGATCTTTATGTGCCCCTCCTTCAGGTTTTGGCGTC	0	0	0	0.0069	0.0424	0.0752	2
T72	Theo1	CTATCGCTTGTGAGGCTCGATCTTTATGTGCCCCTCCTCCAGGTTTGGCGTCT	0	0	0.0001	0.0031	0.03	0.0465	1
T85	Theo1	CTATCGCTTGTGAGGCTCGATCTTTATGTGCCCCTCCTTCGGGTTTGGCGTCT	0	0	0.0001	0.0046	0.0322	0.0385	1
**T2**	Theo2	CAGGGCAATTTGAGGCTCGATCTAGCGCCGTGCCCTCGCTCAGTTGGCGTCG	0	0.0001	0.0093	0.2888	1.0288	1.4868	0
**T3**	Theo3	GCACCAATCTGAGGCTCGATCGTTTATGTGCCCCGACTCTTTAGGTGGTGG	0	0	0.0059	0.341	1.0824	1.4262	0
T4	Theo4	GTGCAGAATTTGAGGCTCGATCTACAAAACGCCTGCATCCTCTGATCGTGGC	0	0.0001	0.004	0.1284	0.3827	0.672	0
T5	Theo5	GAGTGATATATGAGGCTCGATCATTTACCATACCCTGCTCTGTCTTAGGTGG	0	0.0001	0.0039	0.1131	0.3723	0.6314	0

	DFHBI-1T			Frequency [%]						
	ID	FAMILY	SEQUENCE	Round 2	Round 4	Round 6	Round 8	Round 10	Round 12	PM
6 →	D1	DFHBI1	ATCTATCCGATGAGGCTCGATCCAGTGCTTTTGCTCTCTAGAGTCATTCACGT	0	0	0.0002	0.0009	0.0307	0.2513	0
1 →	D2	DFHBI2	GGAACGGTAATGAGGCTCGATCGAATTAGACCGCCTTGATTCCCTCGTGGCG	0	0	0.0006	0.0033	0.0243	0.0869	0
	D3	DFHBI3	GAAGACCCCTGAGGCTCGATCCATCAAACAGATTCTTCATGATGTCGATGCT	0	0	0	0.0006	0.0122	0.0804	0
	D4	DFHBI4	GGCGCACCCCTGAGGCTCGATCTTGGTTGAAATATGCGCTGCGATGGTCGTC	0	0	0	0.0004	0.008	0.0578	0
	D5	DFHBI5	GCCAACCCCTTGAGGCTCGATCTAGTGTGAGAAACTGGCTTGATGTCTACCCT	0	0	0	0.0006	0.0035	0.0254	0

	HO-Trp-A			Frequency [%]						
	ID	FAMILY	SEQUENCE	Round 2	Round 4	Round 6	Round 8	Round 10	Round 11	PM
7 →	**H1**	HO1	CCGGTATCTATGAGGCTCGATCTTCTTACTACCTTCTCTTCAGGTCGGCGCCT	0.0001	0.0017	0.0091	0.0111	0.1033	1.108	0
4 →	**H2**	HO2	CCACGCTGGTGAGGCTCGATCACGGAGACTTCATTTCCCATTGTTGTGTGGT	0	0.0014	0.0018	0.0103	0.0755	0.586	0
	H3	HO3	CCCAACCTATTGAGGCTCGATCCGCTGTTCTTTGGGTGGCTGTCTCTCCGCCT	0	0	0	0.0017	0.0126	0.0774	0
	H4	HO4	CCACGAATCTTGAGGCTCGATCAGATCTCCCTCGATCTATTCTGTGTGGCGTT	0	0	0	0.0009	0.0098	0.0742	0
	H5	HO5	GCAGCTCTACTGAGGCTCGATCTTTATCTTGCTATGCATTTCTAGCCCCTCCT	0	0	0.0018	0.0009	0.0171	0.0615	0

	m6A			Frequency [%]						
		FAMILY	SEQUENCE	Round 2	Round 4	Round 6	Round 8	Round 10	Round 12	PM
4 →	**m1**	m6A1	CCACGCTGGTGAGGCTCGATCACGGAGACTTCATTTCCCATTGTTGTGTGGT	0	0.0009	0.0058	0.1245	0.7344	1.8388	0
	m3	m6A1	CCACGCTGGTGAGGCTCGATCACGGAGACTTCATCTCCCATTGTTGTGTGGT	0	0.0001	0.0005	0.0386	0.2769	0.7538	1
	m17	m6A1	CCACGCTGGTGAGGCTCGATCATGGAGACTTCATTTCCCATTGTTGTGTGGT	0	0	0	0.0036	0.0173	0.0473	1
7 →	**m2**	m6A2	CCGGTATCTATGAGGCTCGATCTTCTTACTACCTTCTCTTCAGGTCGGCGCCT	0	0	0.0014	0.0305	0.3374	0.7844	0
1 →	**m4**	m6A4	GGAACGGTAATGAGGCTCGATCGAATTAGACCGCCTTGATTCCCTCGTGGCG	0	0.0009	0.0033	0.0578	0.1686	0.3329	0
	m79	m6A4	GGAACGGTAATGAGGCTCGATCGAATTAGACCGCCTTGGTTCCCTCGTGGCG	0	0	0	0.0039	0.0083	0.0128	1
3 →	**m5**	m6A5	GGGATGTCGTGAGGCTCGATCAATGTATTTTGCTCCCTGCTCGGTGGTTCCT	0	0	0.0019	0.0328	0.1462	0.3138	0

	Glucose			Frequency [%]						
		FAMILY	SEQUENCE	Round 2	Round 4	Round 6	Round 8	Round 10	Round 11	PM
	**G1**	Glu1	CCACGCTGGTGAGGCTCGATCACGGAGACTTCATTTCCCATTGTTGTGTGGT	0	0.0001	0.0033	0.0508	0.2121	0.409	0
	G9	Glu1	CCACGCTGGTGAGGCTCGATCACGGAGACTTCATTTCCCATTGTTTGTGTGG	0	0	0.0002	0.0035	0.0153	0.0304	2
	**G2**	Glu2	CCACTTCGGATGAGGCTCGATCAACTCCCGTTATAGTCTGGGCAATCGCTGG	0	0.0005	0.0205	0.1449	0.2636	0.3108	0
	G58	Glu2	CCACTTCGGATGAGGCTCGATCAACTCCCATTATAGTCTGGGCAATCGCTGG	0	0	0.0004	0.003	0.0057	0.0075	1
	G87	Glu2	CCACTTCGGATGAGGCTCGATCAACTCCCGTTATAGTCTTGGCAATCGCTGG	0	0	0.0003	0.0013	0.0031	0.0038	1
3 →	**G3**	Glu3	GGGATGTCGTGAGGCTCGATCAATGTATTTTGCTCCCTGCTCGGTGGTTCCTT	0	0.0018	0.0291	0.1805	0.2679	0.3061	0
	G78	Glu3	GGGATGTCGTGAGGCTCGATCAATGTATTTTGCTCCTGCTCGGTGGTTCCTT	0	0	0.0005	0.0029	0.0047	0.0057	2
	G85	Glu3	GGATGTCGTGAGGCTCGATCAATGTATTTTGCTCCCTGCTCGGTGGTTCCTT	0	0	0.0003	0.0016	0.0031	0.0041	2
	G90	Glu3	GGGATGTCGTGAGGCTCGGTCAATGTATTTTGCTCCCTGCTCGGTGGTTCCT	0	0	0.0002	0.0014	0.002	0.0028	1
	G4	Glu4	ACACCTGAATTGAGGCTCGATCTTATTAACACCTCATCAGGAACAACACCCG	0	0	0.0002	0.0014	0.0429	0.203	0
	G5	Glu5	GCTATCTAGTTGAGGCTCGATCAGTGTACCATACTTTCTTCTCCCACCGGCA	0	0.0001	0.0007	0.0095	0.0496	0.0979	0
	G13	Glu5	GCTATCTAGTTGAGGCTCGATCAGTTTACCATACTTTCTTCTCCCACCGGCA	0	0	0.0002	0.0014	0.0097	0.0232	1
6 →	**G6**	Glu6	ATCTATCCGATGAGGCTCGATCCAGTGCTTTTGCTCTCTAGAGTCATTCACGT	0	0.0013	0.0368	0.5822	0.149	0.0586	0
	S18	Glu6	ATCTATCCGATGAGGCTCGATCCAGTGCCTTTGCTCTCTAGAGTCATTCACGT	0	0	0.0003	0.0042	0.0145	0.0192	1
	S96	Glu6	GTCTATCCGATGAGGCTCGATCCAGTGCTTTTGCTCTCTAGAGTCATTCACGT	0	0.0001	0.0004	0.0093	0.0019	0.0009	1
	S98	Glu6	CTATCCGATGAGGCTCGATCCAGTGCTTTTGCTCTCTAGAGTCATTCACGT	0	0	0.0004	0.0062	0.0019	0.0007	4
	S99	Glu6	ATCTATCCGATGAGGCTCGATCCAGTGCTTTGCTCTCTAGAGTCATTCACGT	0	0	0.0004	0.0063	0.0016	0.0006	2
	S100	Glu6	ATCTATCTGATGAGGCTCGATCCAGTGCTTTTGCTCTCTAGAGTCATTCACGT	0	0	0.0002	0.0029	0.0009	0.0005	1

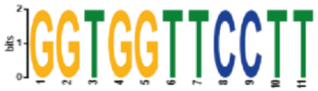

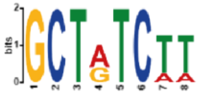

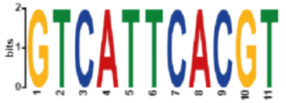

	5-IP7			Frequency [%]						
		FAMILY	SEQUENCE	Round 2	Round 4	Round 6	Round 8	Round 10	Round 12	PM
1 →	I1	IP1	GGAACGGTAATGAGGCTCGATCGAATTAGACCGCCTTGATTCCCTCGTGGCGT	0.0021	0.0043	0.0547	0.297	1.8403	9.1016	0
	I9	IP1	GGAACGGTAATGAGGCTCGATCGAATTAGACCGCCTTGATTCCCTCGTGGCGG	0	0	0.0002	0.0042	0.0265	0.0791	1
	I14	IP1	GGAACGGTAATGAGGCTCGATCGAATTAGACCGCCTTGATCCCCTCGTGGCGT	0	0	0.0002	0.0008	0.0064	0.0456	1
	I18	IP1	GGAACGGTAATGAGGCTCGATCGAATTAGACCGCCTTGATTCCCTCATGGCGT	0	0	0.0006	0.0013	0.0123	0.0417	1
	I19	IP1	GGAACGGTAATGAGGCTCGATCGAATTAGACCGCCTTGATTCCCTCGCGGCGT	0	0	0.0003	0.0006	0.0079	0.0413	1
	I24	IP1	GGAACGGTAATGAGGCTCGATCGAATTAGACCGCCTTGACTCCCTCGTGGCGT	0	0	0.0001	0.0013	0.0055	0.0348	1
	...	IP1								
	I67	IP1	GGAACGGTAATGAGGCTCGATCGATTTAGACCGCCTTGATTCCCTCGTGGCGT	0	0.0001	0.0001	0.0008	0.007	0.0153	1
	**I2**	IP2	CACGAGTTAATGAGGCTCGATCCAGCATGTCCTCTTCATCGTTTGTGGTGTG	0	0	0.0017	0.0228	0.0732	0.1864	0
	**I3**	IP3	CTATCAGGTCTGAGGCTCGATCCCTTGGCAGGTGTTCACATCCGGTTCTTGG	0	0	0.0001	0.0055	0.0302	0.125	0
	**I4**	IP4	CTATCAGGTCTGAGGCTCGATCCGTCAATTGGTCGTGTGCGAGTGTCTACGG	0	0	0.0001	0.001	0.0195	0.1234	0
	**I5**	IP5	ACGAACGGCATGAGGCTCGATCTACGTTACCCCTCTGACGTTGGCATTTGTG	0	0	0.0006	0.0058	0.0385	0.1052	0
	I6	IP6	CAGGGCAATTTGAGGCTCGATCTAGCGCCGTGCCCTCGCTCAGTTGGCGTCGT	0.0001	0.0009	0.0115	0.0053	0.0767	0.1016	0

	ppGpp			Frequency [%]						
		FAMILY	SEQUENCE	Round 2	Round 4	Round 6	Round 8	Round 10	Round 12	PM
1 →	P1	ppGpp1	GGAACGGTAATGAGGCTCGATCGAATTAGACCGCCTTGATTCCCTCGTGGCGT	0.0016	0.036	0.6512	5.1331	24.1979	48.9888	0
	P3	ppGpp1	GGAACGGTAATGAGGCTCGATCGAATTAGACCGCCTTGATTCCCTCGTGGCGG	0.0001	0.0002	0.0091	0.1095	0.1173	0.7745	1
	P6	ppGpp1	GGAACGGTAATGAGGCTCGATCGAATTAGACCGCCTTGATTCCCTCATGGCGT	0	0.0012	0.0046	0.0135	0.1301	0.301	1
	P12	ppGpp1	GGAACGGTAATGAGGCTCGATCGAATTAGACCGCCTTGATCCCCTCGTGGCGT	0	0	0.0016	0.0175	0.0885	0.2069	1
	P13	ppGpp1	GGAACGGTAATGAGGCTCGATCGAATTAGACCGCCTTGATTCCCTCGTGGTGT	0	0	0.0038	0.0232	0.0867	0.1791	1
	P14	ppGpp1	GGAACGGTAATGAGGCTCGATCGAATTAGACCGCCTTGACTCCCTCGTGGCGT	0	0	0.0006	0.0129	0.08	0.173	1
	...	ppGpp1								
	P96	ppGpp1	GGAACGGTAATGAGGCTCGATCGAATTAGACCGCCTTGATTCCCTCGTGTCGT	0	0	0.0008	0.002	0.0114	0.0178	1
4 →	P2	ppGpp2	CCACGCTGGTGAGGCTCGATCACGGAGACTTCATTTCCCATTGTTGTGTGGT	0	0.0002	0.004	0.0514	0.2318	0.907	0
	P8	ppGpp2	CCACGCTGGTGAGGCTCGATCACGGAGACTTCATCTCCCATTGTTGTGTGGT	0	0	0.0008	0.0115	0.0612	0.2281	1
	P4	ppGpp4	CAGGGCAATTTGAGGCTCGATCTAGCGCCGTGCCCTCGCTCAGTTGGCGTCGT	0.0003	0.0016	0.1984	0.8165	2.1538	0.6938	0
	P101	ppGpp4	CAGGGCAATTTGAGGCTCGATCTAGCGCCGTGCCCTCACTCAGTTGGCGTCGT	0	0	0.0014	0.0058	0.0131	0.0083	1
	**P5**	ppGpp5	CACGAACCAATGAGGCTCGATCATGGATCCTTCGTCGAACCTGCTGGCGTTG	0	0	0.0022	0.028	0.1791	0.4992	0
	**P7**	ppGpp7	CAGGAACGGTTGAGGCTCGATCAGATACCCCTCGCTACAATGCGGCATTGGG	0	0	0.002	0.0171	0.0861	0.2846	0
	**P9**	ppGpp9	CATCCACACATGAGGCTCGATCCATTATCATGGCCTGACTCGTAGTCCTGCG	0	0	0.001	0.0059	0.0496	0.2084	0
	**P18**	ppGpp18	CTATCAGGTCTGAGGCTCGATCTTCAGTGTGGTAACGCATCCGTTTGTGCGG	0	0	0.0004	0.0095	0.0169	0.1168	0

Riboflavin			Frequency [%]					
ID	FAMILY	SEQUENCE	Round 2	Round 4	Round 6	Round 9	Round 10	PM
**R1**	Rib1	GTTAGGCTATTGAGGCTCGATCGAAGAAGGAACACACATCTGTCGCTCTGCT	0	0.0005	0	0.2798	5.3896	0
**R2**	Rib2	GAGGTACGTATGAGGCTCGATCGGAAGGCTGGTCTAGGTCCCGTCCTGACCG	0.0001	0.0001	0	0.0227	5.2139	0
**R3**	Rib3	GTCGGAGGTATGAGGCTCGATCACGAAGAAGGCCGATTGGGTTACCCTCCTT	0	0.0002	0	0.1351	2.9985	0
R4	Rib4	AGGCGGAAGATGAGGCTCGATCAAGGTAGGCGGTAGAATCTGTCTTCTCGAT	0	0.0001	0	0.0091	1.6036	0
R5	Rib5	GACTTAGCGTGAGGCTCGATCGAAACGGCTATCATTGAGATACTTGCCCCGT	0	0.0004	0	0.1202	1.3797	0

	S-(2-succinyl) cysteine			Frequency [%]					
	ID	FAMILY	SEQUENCE	Round 2	Round 4	Round 6	Round 8	Round 10	PM
1 →	**S1**	SC1	GGAACGGTAATGAGGCTCGATCGAATTAGACCGCCTTGATTCCCTCGTGGCG	0	0.0003	0.0191	0.1124	0.2749	0
	S31	SC1	GGAACGGTAATGAGGCTCGATCGAATTAGACCGCCTTGATTCCCTCGTGGTG	0	0	0.0007	0.0044	0.0083	1
	S47	SC1	GGAACGGTAATGAGGCTCGATCGAATTAGACCGCTTTGATTCCCTCGTGGCG	0	0	0.0007	0.0031	0.0066	1
3 →	**S2**	SC2	GGGATGTCGTGAGGCTCGATCAATGTATTTTGCTCCCTGCTCGGTGGTTCC	0	0.0022	0.0456	0.2013	0.2227	0
	S72	SC2	AGGATGTCGTGAGGCTCGATCAATGTATTTTGCTCCCTGCTCGGTGGTTCC	0	0	0.0008	0.0034	0.0045	1
	S78	SC2	GGGATGTCGTGAGGCTCGATCAATGTATTTTTGCTCCCTGCTCGGTGGTTC	0	0	0.0004	0.0024	0.0036	4
	**S3**	SC3	CCCAACCTATTGAGGCTCGATCCGCTGTTCTTTGGGTGGCTGTCTCTCCGC	0	0.0006	0.0108	0.0724	0.1515	0
	**S4**	SC4	CTATCGCTTGTGAGGCTCGATCTTTATGTGCCCCTCCTTCAGGTTTGGCGT	0	0.0018	0.0218	0.0138	0.0899	0
	S75	SC4	CTATCGCTTGTGAGGCTCGATCTTTATGTGCCCCTCCTTCAGGTTTGGCGC	0	0.0001	0.001	0.0019	0.0041	1
	S77	SC4	CTATCGCTTGTGAGGCTCGATCTTTATGTGCCCCTCCTTCAGGCTTGGCGT	0	0.0001	0.0008	0.001	0.0036	1
	S5	SC5	GTCCGGCCTCTGAGGCTCGATCTTTCTGGCCTGGCTTAGATACGTGCGCTC	0	0	0.0014	0.0167	0.0787	0

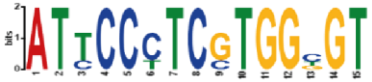

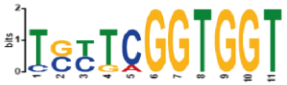

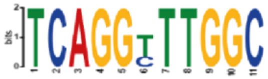

	Cyanocobalamin			Frequency [%]						
	ID	FAMILY	SEQUENCE	Round 2	Round 4	Round 6	Round 8	Round 10	Round 12	PM
	**C1**	Cob1	CCACGCTGGTGAGGCTCGATCACGGAGACTTCATTTCCCATTGTTGTGTGG	0.0001	0.0001	0.0024	0.041	0.2723	1.0699	0
	**C2**	Cob2	GGAGGCTCGATCTTTTAAGTGATTTTATCTCTAGGTCCGCGGTTTCGTGGAT	0.0003	0.0005	0.0025	0.0212	0.1781	0.926	0
	C20	Cob2	GGAGGCTCGATCTTTTAAGTGGTTTTATCTCTAGGTCCGCGGTTTCGTGGAT	0	0	0	0.0011	0.0118	0.0617	1
6 →	**C3**	Cob3	ATCTATCCGATGAGGCTCGATCCAGTGCTTTTGCTCTCTAGAGTCATTCACGT	0.0005	0.0007	0.0554	0.9378	1.7265	0.8026	0
	C19	Cob3	ATCTATTCGATGAGGCTCGATCCAGTGCTTTTGCTCTCTAGAGTCATTCACGT	0.0001	0.0001	0.0058	0.07	0.1223	0.0639	1
	C35	Cob3	ATCTATCCGATGAGGCTCGATCCAGTGCCTTTGCTCTCTAGAGTCATTCACGT	0	0	0.0004	0.0051	0.0297	0.0469	1
	C84	Cob3	ATCTATCCGATGAGGCTCGATCCGGTGCTTTTGCTCTCTAGAGTCATTCACGT	0	0	0	0.0036	0.0144	0.0176	1
	C99	Cob3	ATCTATCCGATGAGGCTCGATCCAGCGCTTTTGCTCTCTAGAGTCATTCACGT	0	0	0.0016	0.0204	0.0223	0.0059	1
	S100	Cob3	GTCTATCCGATGAGGCTCGATCCAGTGCTTTTGCTCTCTAGAGTCATTCACGT	0	0	0	0.0034	0.0089	0.0052	1
1 →	C4	Cob4	GGAACGGTAATGAGGCTCGATCGAATTAGACCGCCTTGATTCCCTCGTGGCG	0	0.0002	0.0068	0.0432	0.2214	0.2666	0

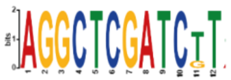

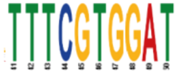

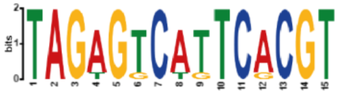

Figure S14. NGS analysis of selections targeting different small molecules. First, 100 the most enriched sequences were sorted in different families with no more than 5-point mutations (PM). Here, only up to first six families are shown or in the case of ppGpp target up to 18 families. Next, different motifs were searched using MEME suite tool for motif discovery.[1] Through different selections, common sequences were found highlighted with the same colour. The common sequences shown in **Supp. Fig. 13** are marked with an arrow and corresponding number.

With the bold, sequences are depicted, which were tested for binding to corresponding target. DFHBI-1T: 3,5-Difluoro-4-hydroxybenzylidene imidazolinone-1T,[2] HO-Trp-A: D-Tryptophanyl Adenosine 5'-Monophosphate Phosphoramidate,[3] m6A: N6-Methyladenosine, 5-IP7: 5-diphospho-*myo*-inositol pentakisphosphate,[4] ppGpp: Guanosine pentaphosphate.

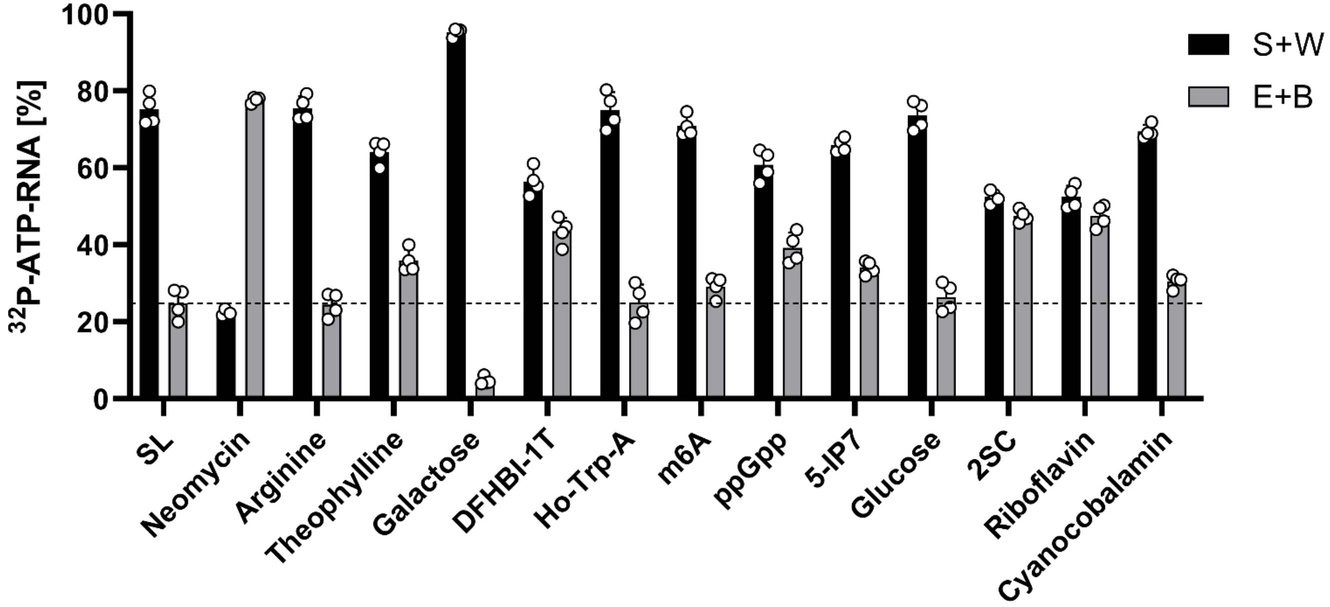

Figure S15. Annealing efficiency of the starting library (SL) and enriched pools selected for different small molecule targets. Annealing efficiency of some enriched pools increases (e.g. neomycin B, theophylline, DFHBI-1T, ppGpp, 2SC, riboflavin), while for mostly failed selections it decreases (e.g. galactose). Error bars show standard deviation (n=2). S+W: Sum of supernatant and three washing fractions, E+B: Sum of elution and beads fraction. DFHBI-1T: 3,5-Difluoro-4-hydroxybenzylidene imidazolinone-1T,[2] HO-Trp-A: D-Tryptophanyl Adenosine 5'-Monophosphate Phosphoramidate,[3] m6A: N6-Methyladenosine, 5-IP7: 5-diphospho-*myo*-inositol pentakisphosphate,[4] ppGpp: Guanosine pentaphosphate, 2SC: S-(2-succinyl) cysteine.

Table S2. List of utilized sequences

Sequence	‘5-‘3
**C2 library**	GGGAGAGGAGGGAGAUAGAUAUCAA-N40-UUUCGUGGAUGCCACAGGAC
**UFZ library**	GGGAGAGGAGGGAGAUAGAUAUCAA-N10-UGAGGCUCGAUC-N30-UUUCGUGGAUGCCACAGGAC
**Forward primer**	AATTCTAATACGACTCACTATAGGGAGAGGAGGGAGATAGATATCAA
**Reverse primer**	GTCCTGTGGCATCCACGAAA
**Bio-ODN**	Biotin-18spacer-GATCGAGCCTCA
**Cy3-ODN_12nt**	Cy3-GATCGAGCCTCA
**Cy3-ODN_11nt**	Cy3-GATCGAGCCTC
**Cy3-ODN_10nt**	Cy3-GATCGAGCCT
**Cy3-ODN_9nt**	Cy3-GATCGAGCC
**Cy3-ODN_8nt**	Cy3-GATCGAGC
**Cy3-ODN_PM**	Cy3-GATCGCGCCTCA
**Forward NGS primer**	6nt index-AGGGAGATAGATATCAA
**Reverse NGS primer**	6nt index-TGGCATCCACGAAA
**HDB1**	GGGAGAGGAGGGAGATAGATATCAAACCCCATCTTGAGGCTCGATCGGCTCGTTTTTCAGGGATAAGTAGATTTAGTTTTTCGTGGATGCCACAGGAC
**HDB2**	GGGAGAGGAGGGAGATAGATATCAAACCGGCACCTGAGGCTCGATCTTTGTTTGCAAAGGGCATTGCATCGATGTGTTTTTCGTGGATGCCACAGGAC
**LM1**	GGGAGAGGAGGGAGATAGATATCAACACTTGAACGTGAGGCTCGATCTTTTTAGCTTTTTACTGCGGCGTTAACGGTTTTCGTGGATGCCACAGGAC
**LM2**	GGGAGAGGAGGGAGATAGATATCAAACTAACAGTCTGAGGCTCGATCTTTTGCAGAAACTTTCGTCTTTTGGCTATCTTTCGTGGATGCCACAGGAC
**LR1**	GGGAGAGGAGGGAGATAGATATCAAACTAAACACTTGAGGCTCGATCTTTATTGTATTTACACCATCTTGTGCATCATTTCGTGGATGCCACAGGAC
**LR2**	GGGAGAGGAGGGAGATAGATATCAATTACCACGCTTGAGGCTCGATCTTTTGCTTTGTCCATCTAGTGTTGTCTGCGTTTCGTGGATGCCACAGGAC
**T1**	GGGAGAGGAGGGAGATAGATATCAACTATCGCTTGTGAGGCTCGATCTTTATGTGCCCCTCCTTCAGGTTTGGCGTCTTTCGTGGATGCCACAGGAC
**T2**	GGGAGAGGAGGGAGATAGATATCAACAGGGCAATTTGAGGCTCGATCTAGCGCCGTGCCCTCGCTCAGTTGGCGTCGTTTCGTGGATGCCACAGGAC
**A1**	GGGAGAGGAGGGAGATAGATATCAACCACTTCGGATGAGGCTCGATCAACTCCCGTTATAGTCTGGGCAATCGCTGGTTTCGTGGATGCCACAGGAC
**I5**	GGGAGAGGAGGGAGATAGATATCAAACGAACGGCATGAGGCTCGATCTACGTTACCCCTCTGACGTTGGCATTTGTGTTTCGTGGATGCCACAGGAC
**P7**	GGGAGAGGAGGGAGATAGATATCAACAGGAACGGTTGAGGCTCGATCAGATACCCCTCGCTACAATGCGGCATTGGGTTTCGTGGATGCCACAGGAC
**G1**	GGGAGAGGAGGGAGATAGATATCAACCACGCTGGTGAGGCTCGATCACGGAGACTTCATTTCCCATTGTTGTGTGGTTTTCGTGGATGCCACAGGAC
**S1**	GGGAGAGGAGGGAGATAGATATCAAGGAACGGTAATGAGGCTCGATCGAATTAGACCGCCTTGATTCCCTCGTGGCGTTTCGTGGATGCCACAGGAC
**R1**	GGGAGAGGAGGGAGATAGATATCAAGTTAGGCTATTGAGGCTCGATCGAAGAAGGAACACACATCTGTCGCTCTGCTTTTCGTGGATGCCACAGGAC
**R2**	GGGAGAGGAGGGAGATAGATATCAAGAGGTACGTATGAGGCTCGATCGGAAGGCTGGTCTAGGTCCCGTCCTGACCGTTTCGTGGATGCCACAGGAC
**R3**	GGGAGAGGAGGGAGATAGATATCAAGTCGGAGGTATGAGGCTCGATCACGAAGAAGGCCGATTGGGTTACCCTCCTTTTTCGTGGATGCCACAGGAC
**C1**	GGGAGAGGAGGGAGATAGATATCAACCACGCTGGTGAGGCTCGATCACGGAGACTTCATTTCCCATTGTTGTGTGGTTTTCGTGGATGCCACAGGAC

Reference

[1] T. L. Bailey, J. Johnson, C. E. Grant, W. S. Noble. "The MEME suite" *Nucleic acids research* **2015**, *43*, W39-W49.

[2] G. S. Filonov, J. D. Moon, N. Svensen, S. R. Jaffrey. "Broccoli: rapid selection of an RNA mimic of green fluorescent protein by fluorescence-based selection and directed evolution" *J Am Chem Soc* **2014**, *136*, 16299-16308.

[3] O. Doppleb, J. Bremer, M. Bechthold, C. Sánchez Rico, D. Göhringer, H. Griesser, C. Richert. "Determining the Diastereoselectivity of the Formation of Dipeptidonucleotides by NMR Spectroscopy" *Chemistry–A European Journal* **2021**, *27*, 13544-13551.

[4] I. Pavlovic, D. T. Thakor, J. R. Vargas, C. J. McKinlay, S. Hauke, P. Anstaett, R. C. Camuña, L. Bigler, G. Gasser, C. Schultz. "Cellular delivery and photochemical release of a caged inositol-pyrophosphate induces PH-domain translocation in cellulo" *Nature communications* **2016**, *7*, 10622.
